# rs67047829 genotypes of *ERV3-1/ZNF117* are associated with lower body mass index in the Polish population

**DOI:** 10.1038/s41598-023-43323-3

**Published:** 2023-10-10

**Authors:** Jeremy S. C. Clark, Konrad Podsiadło, Marta Sobalska-Kwapis, Błażej Marciniak, Kamila Rydzewska, Andrzej Ciechanowicz, Thierry van de Wetering, Dominik Strapagiel

**Affiliations:** 1 Department of Clinical and Molecular Biochemistry, Pomeranian Medical University, al. Powstańców Wlkp. 72, 70-111 Szczecin, Zachodniopomorskie, Poland; 2 Biobank Lab, Department of Oncobiology and Epigenetics, Faculty of Biology and Environmental Protection, University of Łodż, 90-237 Łódż, Poland

**Keywords:** Genetics, Physiology, Biomarkers, Diseases, Endocrinology, Medical research

## Abstract

There is now substantial evidence that zinc-finger proteins are implicated in adiposity. Aims were to datamine for high-frequency (near-neutral selection) pretermination-codon (PTC) single-nucleotide polymorphisms (SNPs; n = 141) from a database with > 550,000 variants and analyze possible association with body mass index in a large Polish sample (n = 5757). BMI was regressed (males/females together or separately) against genetic models. Regression for rs67047829 uncovered an interaction-independent association with BMI with both sexes together: mean ± standard deviation, kg/m^2^: [G];[G], 25.4 ± 4.59 (n = 3650); [G](;)[A], 25.0 ± 4.28 (n = 731); [A];[A], 23.4 ± 3.60 (n = 44); additive model adjusted for age and sex: p = 4.08 × 10^–5^; beta: − 0.0458, 95% confidence interval (CI) − 0.0732 : − 0.0183; surviving Bonferroni correction; for males: [G];[G], 24.8 ± 4.94 (n = 1878); [G](;)[A], 24.2 ± 4.31 (n = 386); [A];[A], 22.4 ± 3.69 (n = 23); p = 4.20 × 10^–4^; beta: − 0.0573, CI − 0.0947 : − 0.0199. For average-height males the difference between [G];[G] and [A];[A] genotypes would correspond to ~ 6 kg, suggesting considerable protection against increased BMI. rs67047829 gives a pretermination codon in *ERV3-1* which shares an exonic region and possibly promoter with *ZNF117*, previously associated with adiposity and type-2 diabetes. As this result occurs in a near-neutral Mendelian setting, a drug targetting *ERV3-1/ZNF117* might potentially provide considerable benefits with minimal side-effects. This result needs to be replicated, followed by analyses of splice-variant mRNAs and protein expression.

## Introduction

### Pretermination codons

Most pretermination codons (PTCs) arise from single nucleotide polymorphisms (SNPs) which give a translation stop codon: TGA, TAG or TAA. Premature termination of translation often leads to a truncated protein unable to fulfill its function, might result in mRNA retention in the nucleus^1^, and promotes mRNA instability via the nonsense-mediated mRNA decay (NMD) pathway^2^.

It is possible, however, that protein function may only be modified rather than eradicated despite pretermination, because functional domain(s) might remain intact and/or if only some splice forms are affected. Theoretically a membrane domain might be eliminated resulting in enhanced and/or altered function of the now water-soluble truncated peptide^3,4^. Possibly occasional read-through might occur, especially as termination can depend on a poly(A) tail as well as a termination codon^5^.

In a genome-wide study by Yngvadottir et al.^6^ pretermination codons were found to be common, many disadvantageous over evolutionary timescales, and some were suggested to have beneficial effects. Pretermination codons are so prevalent in the human population that MacArthur et al.^7^ in 2012 suggested widespread genome redundancy in order to cope with loss of function of particular proteins. They estimated that a typical genome contains around 20 genes completely inactivated, presumably compensated by other proteins with similar function (note they also suggested further validation is needed for some loss-of-function mutations).

In most cases it is expected that pretermination results in loss of protein function which might then be associated with disease. MacArthur et al.^7^ identified 26 recessive disease-causing mutations associated with severe early-onset conditions, such as Leber congenital amaurosis, harlequin ichthyosis, osteogenesis imperfecta and Tay-Sachs disease, and 20 strong candidates for dominantly-inherited disease, including adult-onset muscular dystrophy, Charcot-Marie-Tooth disease and mucolipidosis. They also predicted that loss-of-function might be associated with the risk of common, complex diseases such as Crohn’s disease and rheumatoid arthritis.

Some examples where pretermination confers positive effects have been found e.g. carriers of a *CASP12* stop allele are more resistant to severe sepsis^8^, and an *ACTN3* stop allele has been associated with increased athletic endurance^9,10^.

### Obesity

The genetics of obesity are extremely complex. It is thought that genetics could contribute up to 70% of obesity risk^11^ and > 100 genetic variants have been found to contribute, with most strongly influenced by an obesogenic environment i.e. the presence of obesity-related genetic combinations does not necessarily result in obesity without such an environment. (There are also forms of childhood monogenic obesity.)

A genome-wide association study by Albuquerque et al.^12^ found that the *FTO* gene, encoding the alpha-ketoglutarate-dependent dioxygenase FTO protein, had a strong influence on polygenic obesity susceptibility, possibly affecting food intake and energy expenditure ratio^13^. *FTO* intronic variants (linkage disequilibrium block 8) were strongly associated with overweight in males only^14^. Other genes associated with obesity include *MC4R* (synergistic with *FTO*)*;* the leptin gene and receptor, and genes encoding ectoenzyme nucleotide pyrophosphate phosphodiesterase 1, tumor necrosis factor alpha, interleukin-6, peroxisome proliferator–activated receptor gamma, angiotensin-converting enzyme, and glutathione S-transferase^11^. These are thought to influence one or more of: "food intake control, appetite behaviour, energy balance, insulin signalling, glucose and lipid metabolism, adipocyte (…) differentiation, and metabolic disorders"^11^. Muller et al.^15^ have argued that obesity is such a complex phenotype that studies should concentrate on more specific phenotypes such as one or more in this list, but Speakman et al.^13^ have counter-argued that an increase in sample numbers might well still provide insights into obesity. Albuquerque et al.^12^, in 2017, suggested that the genetic variants found till then contributed only a small percentage of the total estimated body mass index (BMI) heritability (assumed to be closely related to obesity risk), and in any case probably cannot account for the rapid spread of obesity. Therefore, although new associated SNPs have been found since then (e.g. see Sobalska-Kwapis et al.^14^), a search for further genetic variants is warranted.

As many pretermination codons have been found to affect metabolism^16^ it is possible that association might be found between the presence of one or more pretermination codons and body mass index.

### High-frequency PTC-SNPs

Fujikura^16^ identified 246 PTCs, from an initial number of 16,281 segregating PTCs, where the new alleles have risen to high frequencies (between 1 and 96%), and these formed the basis for the present study for the following reasons: (a) It can be presumed that those with > 1% minor allele frequency (MAF) were subject to near-neutral or "slightly deleterious" selection (Zhang and Li^17^ defined SNPs with < 5% MAF as at least being "slightly deleterious") and, as the genetic contribution to obesity risk is considerable and overweight/obesity affects large percentages of the population, high-frequency SNPs (especially functional SNPs such as PTC-SNPs) might well contribute; (b) Approximately half (125/246) of these PTC-SNPs were clearly distributed among 15 biological processes: olfaction (n = 32), zinc fingers (15), spermatogenesis (15), keratins (15), immunoglobulins (8), immune defense receptors (6), taste (6), drug metabolism (6), solute carrier genes (5), RNA viruses (4), melanoma-associated antigens (3), acyl-CoA synthetase medium chains (3), ligands for NGK2D (3), POTE ankyrin domains (2), and interferons (2). The other half (n = 121) were not classified, overall showing that high-frequency PTC-SNPs are distributed in many biological processes, some of which are connected with overweight or obesity (e.g. olfaction^18^, taste^18^, zinc-finger transcription factors, metabolism, fatty-acid synthesis, and others). (c) Some of these processes appear to be related (and also to obesity), perhaps indicating reduced selection contraints as a common factor.

As most contributing genetic factors to the risk for overweight/obesity are postulated as yet to be discovered, it was decided to assess as many of these high-frequency PTC-SNPs as could be found in a large exomic SNP database (“POPULOUS”) with data collected from the Polish population.

### Alternative linkage

Although the class of SNPs which give pretermination codons is unusual in the fact that many cause functional changes to proteins, it is also possible that any SNP can be linked with more than one gene, and possible functional effects on a second gene might be more difficult to predict. Linkage to all genes (from the dbSNP database) for 139 high-frequency PTC-SNPs is shown in Supplementary Table S1.

For rs67047829, which forms a PTC in *ERV3-1* and which was analyzed further, a linked second gene was identified: the classic zinc finger protein gene *ZNF117*, partially frame-shifted from *ERV3-1*. This SNP was found in a proposed *ZNF117* regulatory region (see “Discussion”), has an exonic region in common, and possibly shares a promoter region with *ERV3-1*.

A classic (= Cys2His2-like) zinc finger is a peptide structural motif stabilized by zinc with a beta-pleat, beta-pleat, alpha-helix, often with DNA-binding ability. Zinc-finger proteins are therefore often transcription factors, act as intranuclear hormone receptors and have been implicated (including *ZNF117*) in obesity and in regulating adipogenesis^19^. For example, a review of homocysteine and obesity suggested that homocysteine acts via Zfp407 to upregulate adipogenesis and change fatty-acid metabolism, leading to obesity predisposition^20^. In mice, a partial deficiency in Zfp217 resisted diet-induced obesity and increased energy metabolism^21^.

### Aim

The aim of the present study was to datamine for possible association between BMI and high-frequency pretermination codons found in a large exomic SNP database (“POPULOUS”), with data collected from the entire geographical region of the Polish population. The POPULOUS database contained over 550,000 SNPs each from 5757 subjects who declared themselves healthy. The hypothesis was that a high-frequency pretermination codon might be found to be associated with body mass index, although linked associations with the regulatory region of a second gene were also possible.

## Material and methods

Access to the POPULOUS database was granted for this study following a signed licence agreement (PUM_UL_001). The POPULOUS database was the outcome of the project TESTOPLEK (funded by the Innovative Economy Operational Programme provided by the European Regional Development Fund 2007–2013), which was approved by the regional ethics committee (the Institutional Review Board of the University of Łódź) and all procedures were in accordance with the latest Declaration of Helsinki. Genetic data was made available from anonymous, Polish, unrelated volunteers, who had declared themselves as healthy and signed written informed consent. Procedures for collecting samples, DNA isolation and genetic analysis can be found in Sobalska-Kwapis et al.^14^. Exome SNP beadchips (HumanCoreExome-24 v1.0 and v1.1; Illumina, San Diego, CA, USA) were used, giving SNP allele values for 551,915 SNPs (around one half of SNPs analyzed by these beadchips are found in exons, the rest in introns or other regions of the genome^14^).

The high-frequency PTC-SNP rs numbers (defined at www.ncbi.nlm.nih.gov/snp) of all 246 SNPs found in Table S1 of the study by Fujikura^16^ were searched for and a total of 141 premature termination codons resulting from single nucleotide polymorphisms were initially identified as having rs numbers in the Illumina beadchip lists.

A full list of all SNP values (including duplicates) for all 5757 subjects is given in Supplementary Table S2. This table also contains data for three SNPs which were analyzed previously by Sobalska-Kwapis et al.^14^ for the same phenotype, BMI, with the same POPULOUS dataset. In Sobalska-Kwapis et al.^14^, several SNPs were found to be associated with BMI, including three *FTO* variants: rs1558902, rs1421085 and rs9939609. The effect sizes for these (from Sobalska-Kwapis et al.^14^) were: rs1558902: beta = 0.349, 95% CI 0.189:0.509, p = 1.93 × 10^–5^; rs1421085: beta = 0.345, 95% CI 0.185:0.505, p = 2.42 × 10^–5^; and rs9939609: beta = 0.312, 95% CI 0.152:0.472, p = 1.33 × 10^–4^. From the actual SNP values for these three SNPs, three power estimations were performed using our Monte-Carlo Kruskal–Wallis power tool^22^ (see Supplementary File S5) which gave estimated power of 85.5%, 86.6% and 82.1%, respectively.

Two PTC-SNPs were removed from regression analyses: rs7120775: allele G gives a pretermination codon, but the beadchips analyze alleles C and T; and rs545652: the gene *C17orf77* or *CD300LD* is now recognised as giving anti-sense RNA only and no protein (see www.ncbi.nlm.nih.gov/snp/rs545652 and www.ncbi.nlm.nih.gov/gene/146723). Information concerning the remaining 139 SNPs, including population frequencies and possible effects on expressed proteins, is given in Supplementary Table S1. Two PTC-SNPs, rs497116 and rs35032582, were found to have only one allele (A or C, respectively) for those subjects with BMI data in this study, leaving 137 SNPs for calculations taking into account multiple testing.

Although data is given for the 5757 subjects in Supplementary Table S2, Body mass index (BMI) is missing from 663 subjects and age from a further 44 subjects, giving a total of 5050 subjects for initial regressions. For a further 601 subjects, ≥ 5% SNP values were missing and these were also removed for the final regressions with rs67047829, giving 4425 subjects.

All statistical analyses were performed using the R statistical platform (version 4.2.3, 2023; RRID:SCR_001905, https://cran.r-project.org^23^). Datamining regressions used the R function [SNPassoc_2.1-0] *WGassociation*^24^ for four genetic models (dominant, recessive, heterozygote (= over-dominant) and log-additive) versus BMI: (mass in kg)/(height in m)^2^. For the quantitative phenotype and adjustors given, *WGassociation* was a wrapper for the following R model (verifiable using R *library*(*SNPassoc*), *getAnywhere*(*association.fit*), *getAnywhere*(*intervals.dif*)):

mod0 <- glm(BMI ~ age + sex, family = "gaussian")

mod1 <- glm(BMI ~ genetic_model(SNP) + age + sex, family = "gaussian") 

anova(mod1, mod0, test="F")

Additional comparisons to compute odds ratios were performed (comparing groups 1 + 2 and 3 + 4) using BMI as a categorical variable with four BMI groups as defined by the World Health Organisation: group 1: "underweight", < 18.5 kg/m^2^; group 2: "normal weight", 18.5–24.99 kg/m^2^; group 3: "overweight", 25–29.99 kg/m^2^; group 4: "obese", > 30 kg/m^2^^25^. All preliminary tests could not include adjustments for interactions.

Further, more rigorous regressions (in terms of conforming to assumptions) were performed with rs67047829 (chromosome 7:64,992,360, GRCh38; canonical SPDI: NC_000007.14:64992359:G:A; ENST00000394323.3:c.667C>T, ENSP00000391594.1:p.Arg223Ter) using subjects restricted to those with < 5% missing genotype data and the R models:

glm(genetic_model(SNP) ~ BMI + age + sex, family = binomial, data)

for dominant, recessive or heterozygote genetic models, and

car_3.0-12::Anova(lm(BMI ~ Additive_model(SNP) + age + sex), type = "II", white.adjust = TRUE)

for the additive model, both with or without adjustments and interactions for age and sex. Testing for adjustments with age and sex, and interactions, is important as it is well known for example, and has been shown for the database used, that year of birth (which regresses identically to age) is associated with obesity with increase over time^14^. These analyses were also performed for males and females separately. Diploid SNP bases, i.e. homozygote [A];[A], heterozygote [A](;)[G] and homozygote [G];[G] are abbreviated in this article as AA, AG (or GA) and GG, respectively.

Odds ratios were calculated using R [epitools_0.5–10.1] *oddsratio*^26^, which produces a median-unbiased estimate and uses the mid-p exact method for confidence intervals (CIs). All statistical tests were two-tailed with cut-off defined as p = 0.05, with or without false discovery rate (R [FDRestimation_1.0.1] *p.fdr*^27^), or Bonferroni, correction. Graphics were created or modified using R and/or Mac Preview (version 11; Cupertino, CA, USA) or Inkscape (version 1.3; RRID:SCR_014479, http://www.inkscape.org).

### Ethics approval and consent to participate

Database production was approved by the regional ethics committee (Institutional Review Board of the University of Łódź). Genetic data was from anonymous, healthy, Polish, unrelated volunteers who signed written informed consent (see Sobalska-Kwapis et al.^14^).

## Results

### Characteristics of the POPULOUS sample

The sample consisted of 5757 subjects who declared themselves healthy: 2824 (49.1%) females and 2933 (50.9%) males. According to the Polish Central Statistics Office, (https://demografia.stat.gov.pl/BazaDemografia/StartIntro.aspx), the Polish population consisted of ~ 38,538,400 individuals in the year 2012 and the sample represented ~ 0.015% of the population.

The mean BMI for the final regression sample was 25.3 ± 4.53 kg/m^2^ (Table 1) with, as expected, higher values for females (26.0 ± 4.07 kg/m^2^) than males (24.7 ± 4.84 kg/m^2^).

The numbers of subjects sampled generally decreased with increasing age with a mode at age 32 years old (y.o.) (n = 186; year of birth (y.o.b.) 1980); the smallest number was found at 74 y.o. (n = 26; y.o.b. 1938). Sex proportion at each age was in most cases similar, but at some ages it was disrupted. The largest male over-representation (n = 28; 73.7%) was found at 75 y.o. (y.o.b. 1937), while at 43 y.o. (y.o.b. 1969) the females (n = 66; 60.6%) were over-represented. Overall, mean ages for males (42.3 ± 15.8 y.o.) and females (41.7 ± 15.3 y.o.) were similar (Table 1).

**Table 1:** Body mass index and age of subjects in the subset (n = 4425) extracted from the POPULOUS database, according to sex and rs67047829 genotype, used for the final regressions in which age and sex were adjustors.

Group	Genotype or ALL (all genotypes)	n	Body mass index (kg/m^2^)		Age (years)	
			Median (m.a.d.)	Mean (s.d.)	Median (m.a.d.)	Mean (s.d.)
Both sexes together	ALL	4425	24.8 (2.88)	25.3 (4.53)	41 (12)	42.3 (14.8)
	AA	44	23.3 (3.00)	23.4^a^ (3.60)	38.5 (13)	41.6 (14.9)
	GA	731	24.4 (2.77)	25.0^a^ (4.28)	41 (12)	42.1 (14.7)
	GG	3650	24.9 (2.87)	25.4^a^ (4.59)	41 (12)	42.4 (14.8)
Males	ALL	2287	23.7 (2.94)	24.7 (4.84)	41 (12)	42.7 (14.9)
	AA	23	20.9 (2.90)	22.4^b^ (3.69)	41 (12)	43.0 (14.2)
	GA	386	23.4 (2.90)	24.2^b^ (4.31)	40.5 (11.5)	42.2 (14.7)
	GG	1878	23.8 (2.97)	24.8^b^ (4.94)	41 (12)	42.8 (14.9)
Females	ALL	2138	25.6 (2.59)	26.0 (4.07)	41 (12)	41.9 (14.6)
	AA	21	24.7 (2.48)	24.5 (3.27)	35 (11)	40.0 (15.8)
	GA	345	25.4 (2.42)	25.9 (4.07)	41 (12)	42.0 (14.8)
	GG	1772	25.7 (2.63)	26.1 (4.07)	40.5 (12.5)	42.0 (14.6)

Continuous data to 3 significant figures; integer data to nearest multiple of 0.5

*m.a.d.* median absolute deviation, *s.d.* standard deviation, *n* number of subjects.

^a,b^Significant difference among genotypes: linear regression with additive model.

For each of the 139 high-frequency premature-termination codons resulting from single nucleotide polymorphisms identified in the Illumina exome beadchip lists, BMI information for three possible genotypes is given in Supplementary Table S3 and for rs67047829 is summarized, including according to sex and with age data, in Table 1.

**Figure 1:**
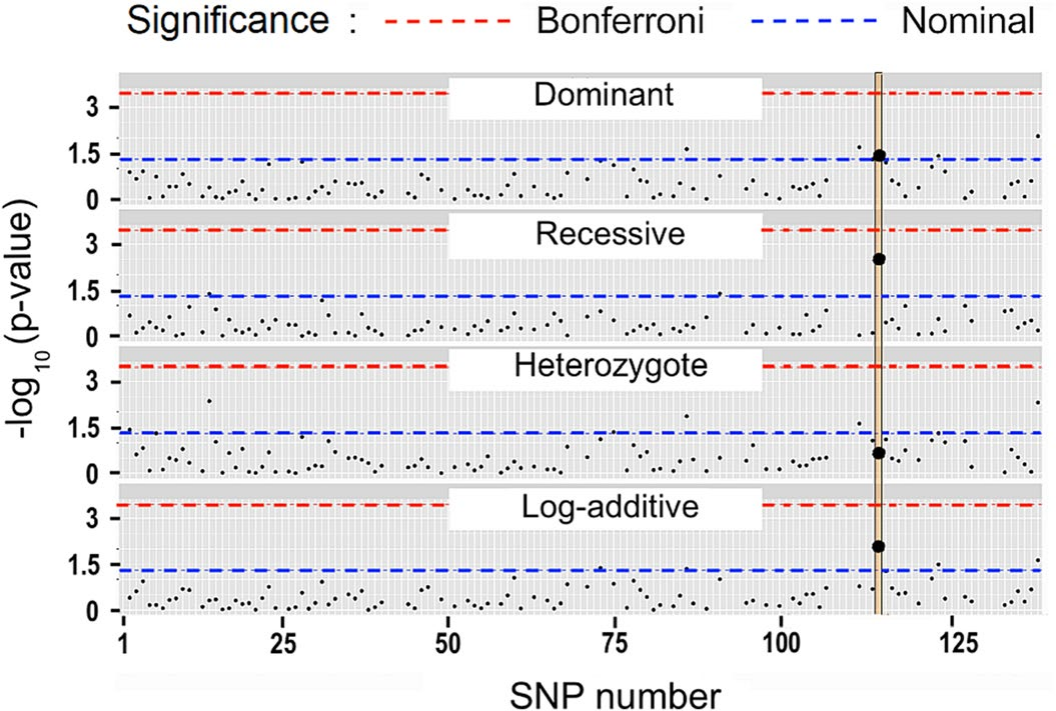
Manhattan plot. Regressions relating four genetic models (heterozygote = over-dominant) for high-frequency pretermination codon (PTC) single-nucleotide polymorphisms (SNPs) with body mass index. P-values for PTC-SNP rs67047829 are shown as large points on vertical black lines. SNP numbers are from the 139 SNPs in Supplementary Table S3. Significance levels are nominal (p = 0.05) or after Bonferroni correction.

Datamining regression results are given in Supplementary Table S3 for 139 PTC-SNPs, and a Manhattan plot is shown in Fig. 1. Several SNPs gave low p values with regressions, but rs67047829, located in *ERV3-1* (Ensembl:ENSG00000213462; Chromosome 7:64,990,356–65,006,687) and linked with *ZNF117* (ENSG00000152926; Chromosome 7:64,971,772–65,006,684), gave the lowest p-value and p < 0.05 with three models (Supplementary Table S3) and was chosen for further study. With this initial trawl individual p-values did not survive false discovery rate correction (this result can be generated from Supplementary file S4).

A more rigorous regression study was conducted with rs67047829 versus BMI using logistic regression for dominant, recessive and heterozygote models, and linear regression for the additive model, with adjustments and/or interactions for sex and age, and with subjects removed with more than 5% missing genotype data (removing 670 subjects). Results are shown in Table 1 and Supplementary file S4 (regressions can also be run without this last criterion; the effects are still highly significant).

**Figure 2:**
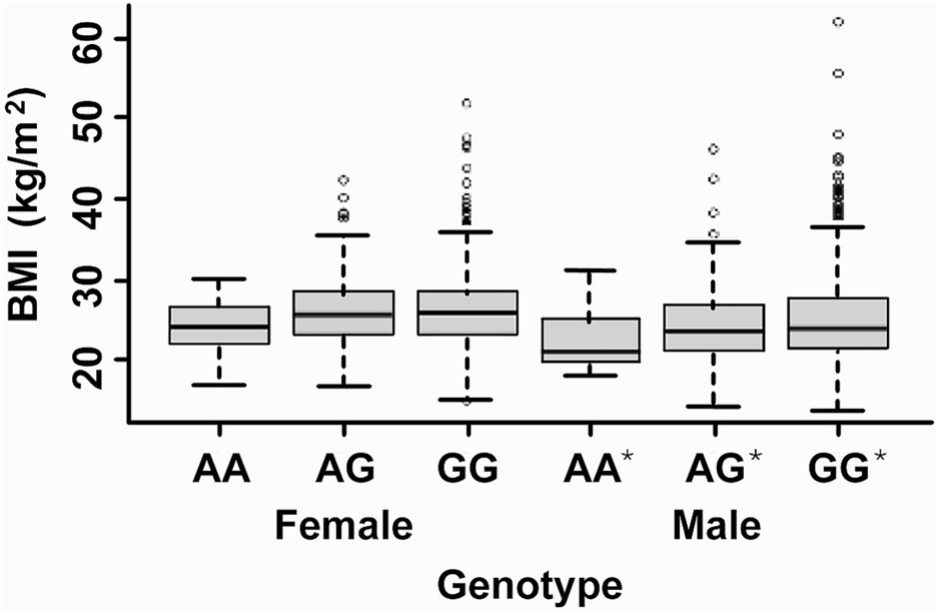
Body mass index (BMI; kg/m^2^) versus genotype for the high-frequency pretermination-codon single nucleotide polymorphism rs67047829. Box plots show medians (m), interquartile range (q_0.25_ to q_0.75_), range between q_0.25_–1.5(m-q_0.25_) to q_0.75_ + 1.5(q_0.75_-m), and outliers. *Statistically significant difference among genotypes with additive model.

Final regression results for males and females together (n = 4425) showed highly statistically-significant, and for males (n = 2287) statistically-significant, associations for dominant (p = 0.00408, p = 0.00854, for both sexes and males, respectively) and recessive (p = 0.00441, p = 0.0243) genetic models, and these were also significant for the heterozygote model (p = 0.0288, p = 0.0359): all of these detected no main effects of age or sex which were therefore removed from the models. The linear additive model was also highly significant for both sexes together: p = 4.08 × 10^–5^; beta: − 0.0458, 95% CI − 0.0732 : − 0.0183 and for males: p = 4.20 × 10^–4^; beta: − 0.0573, CI − 0.0947 : − 0.0199 (Fig. 2); both with adjustments for age and sex but no significant interactions. The latter result for both sexes together survived Bonferroni correction, calculated as p < 0.05/((137 × 4) + (4 × 3)) = 8.93 × 10^–5^.

Corresponding results for females (n = 2138) were not significant (dominant p = 0.233; recessive p = 0.0766; heterozygote p = 0.453; additive p = 0.0652).

## Discussion

Datamining showed that most (80/137 = 58%) of the high-frequency PTC-SNPs, defined as having minor-allele frequencies (MAFs) between 1 and 99%, including rs67047829, had MAFs > 5%, indicating most likely near-neutral selection (Supplementary Table S1).

Initial regression models for several PTC-SNPs versus BMI gave low p-values (Supplementary Table S3), suggesting possible future studies, but only one was studied further as multiple-testing effects are well known. Proportions, probable consequences and initial regression results for all PTC-SNPs are given in Supplementary Tables S1 and S3.

Only the PTC-SNP with the lowest p-value, rs67047829 (recessive model), was further analyzed with final, more rigorous, regression (see Methods). Differences between the initial and final regressions were: for final regressions: (1) subjects with > 5% missing genotypes were removed; (2) statistical models were more advanced: for two-valued genotype models: logistic, instead of linear, regression; for additive model: type III anova and White's estimator with linear regression (in future, if interactions are implemented, a Kruskal–Wallis approximation test such as R [coin] *independence_test*^28^ might be preferred); (3) interactions were assessed; and (4) both sexes were analyzed.

Final rs67047829 regression gave statistically-significant results for all models with both sexes together (n = 4425), indicating the most appropriate model was additive (as this includes aspects of the other three; for a heterozygote result with unbalanced genotype sample sizes). Only one model could be criticized for a low number of subjects: recessive, with AA n = 44 versus GG + GA n = 4331; contrast this with dominant: AA + GA n = 775 versus GG n = 3650.

The significance pattern for both sexes together was repeated with male results (n = 2287) with higher p-values. Female results (n = 2138) were not significant.

Association with the AA genotype was considerable. Regression beta estimates and additional effect sizes: odds ratios of overweight/obese versus normal-weight/underweight, are given in Supplementary file S4. The odds ratio for both sexes together to be overweight/obese with the AA genotype was 0.430 (AA vs. GA + GG; underweight + normal weight vs. overweight + obese; CI 0.234:0.761; p = 0.00324); for males the values were not statistically significant for this comparison (odds ratio 0.492, CI 0.207:1.07, p = 0.0771). From linear additive models the genetic heritability was estimated to be large: adjusted r-squared for both sexes: 0.133; males: 0.168.

The BMIs (kg/m^2^; Table 1; Fig. 2) with both sexes together for AA, GA and GG genotypes were: means (standard deviations, n): 23.4 (3.60, 44), 25.0 (4.28, 731), 25.4 (4.59, 3650), respectively (males only: 22.4 (3.69, 23), 24.2 (4.31, 386), 24.8 (4.94, 1878)) showing large absolute effect sizes, especially between AA and other genotypes.

Therefore, the high-frequency PTC-SNP rs67047829 was found in the present study to be potentially associated with BMI with protection against overweight in both sexes together and in males alone in the Polish population, with the additive model as most appropriate, adjusted for year of birth and sex (with interactions not significant). Female results were not significant (Fig. 2), either indicating reduced association and/or insufficient data.

The final additive-model p-value for both sexes together was 4.08 × 10^–5^, surviving Bonferroni correction despite the large number of models tested. However, it is easy to show (from false-positive prevalence minus 5%) that the major error-type affecting medical-association studies, and possibly all scientific results describing differences between categorically-distinct groups of subjects, is not random error but systematic error, as defined by Norena et al.^29^. In the medical sciences there is overwhelming evidence that result replication, which should involve an (inherent or enhanced) attempt at resetting (unforeseen, known and unknown) systematic-error values, should be an absolute requirement^30–32^. This result should therefore be replicated at another Institute before being used as part of inference for further, different, tested hypotheses.

Further considerations regarding genotyping, phenotype and global prevalence are now described.

### Genotyping

- DNA samples were collected from saliva, likely containing oral microbiome. Although DNA yield is lower from saliva compared to blood^33^, the response rate from subjects is significantly higher with saliva^34^. It is also assumed that the stringent methods, and the length of the beadchip probes, were sufficient to avoid result contamination.
- Even though the majority of SNP-value calls were thought to be accurate, there will still be some errors. In the present study 72/141 (51%) SNPs had no missing genotype data at all (including rs67047829), one indication of successful genotyping. However, to counter theoretical problems with particular subjects, subjects with > 5% missing genotypes were removed for final regressions (note the trade-off between removing subjects and statistical significance).

### Phenotype

(c) BMI is used in the diagnosis of many diseases. However, it does not differentiate between subcutaneous, visceral or abdominal-visceral obesity and precise measurements of these might be productive.
(d) For individuals with average height during this period (~ 1.75 m^35^) the difference between GG and AA genotypes would correspond to ~ 6 kg, of considerable benefit.

### Global prevalence

(e) Global prevalence of the rs67047829:A allele is 9%, meaning that, as for ~ 30% of human non-synonymous SNPs with MAF > 5%, overall effects from this SNP or a linked region are likely near-neutral, even though it potentially alters expressed protein from *ERV3-1* considerably (see below).

The tendency to obesity is thought to be beneficial in environments with chronic or occasional food shortage, conserving energy resources needed later in famine. Pleiotropic effects could therefore be at play and near-neutral selection overall might result from positive effects balanced by negative effects in a food-rich environment.

Although world distribution of the minor allele might reflect diffusion from the location of an original mutation (according to Fujikura^16^ high-frequency PTC-SNPs have a relatively recent origin), it is also possible that balancing effects are partially reflected in the present distribution. The rs67047829 A allele prevalence shows high ethnic dependence: 3–4% in people of relatively-recent African descent, ~ 9% with European descent, 11–12% in Latin America and 14–22% with Asian descent (Alfa version 20201027095038^36^), perhaps partially reflecting food availability (?). (Note that from this, analysis of people with Asian descent might provide easier elucidation of relationships between rs67047829 and obesity/overweight phenotypes.)

### Limitations

1. The sample size, although large, may not have been sufficiently representative of the Polish population; although obesity studies have been successful before with this data^14^. Additionally, to determine the odds ratio for males (and to confirm lack of effects in females), studies would need a larger Polish population sample or to use a different ethnic group.
2. More accurate measurements of body fat could be made using dual-energy X-ray absorptiometry rather than BMI.

### Potential effects on protein expression

**Figure 3:**
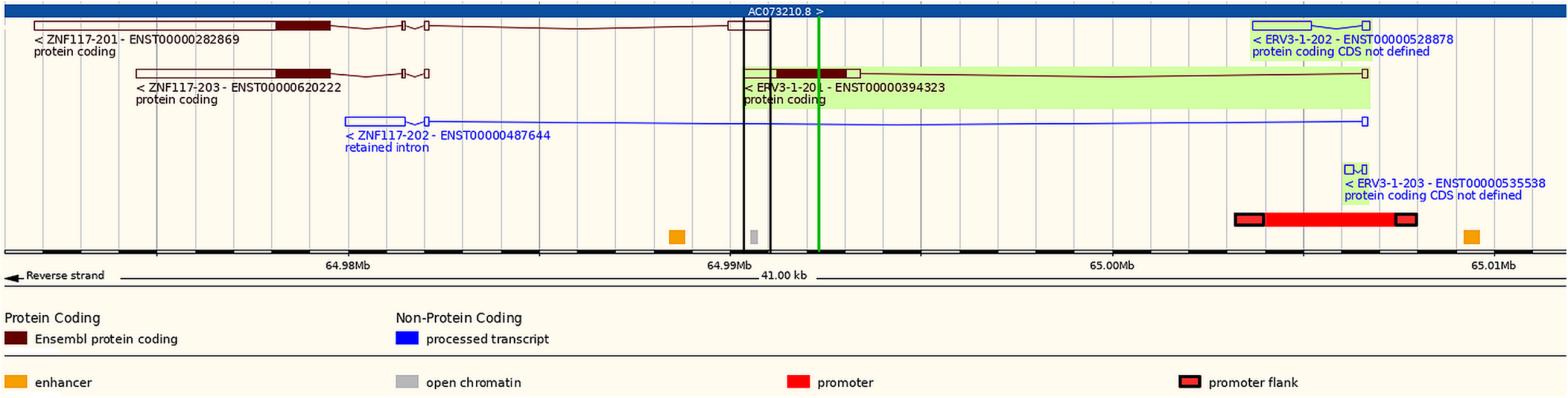
Genetic annotation of *ERV3-1* and *ZNF117*. Chromosome region 7:64,971,000–65,012,000, showing transcript relationships including a promoter region and an overlapping exonic region (between vertical black lines) seen in *ERV3-1*–201 and *ZNF117*-201. The location of rs67047829 is shown as a vertical green line. Genes are reverse strand. Source of image: modified from Ensembl version 109, comprehensive gene set from GENCODE 43^38^.

An association cannot be linked directly to a causal effect. However, it is worth speculating on the possibilities, especially as pretermination codons have direct functional effects on proteins. rs67047829 potentially affects two overlapping, partially-frameshifted, genes encoded in the reverse strand: *ERV3-1* and *ZNF117*, which possibly share a promoter and, additionally, the protein-coding transcript *ZNF117*-201 shares an untranslated exonic region with an untranslated exonic region of *ERV3-1*-201 (Fig. 3).

rs67047829 only gives a PTC in *ERV3-1*, and potentially has considerable effect on protein production from this gene. *ERV3-1* is an evolutionary remnant from a human endogenous retrovirus (HERV) infection, and protein truncation might still have possible biological consequences. However, recent research has provided evidence that *ZNF117* variation is associated with type-2 diabetes and adiposity, and *ZNF117* is discussed first.

### *ZNF117*

According to Kewitz and Staege^37^, several zinc finger protein genes including *ZNF107*, *ZNF138*, and *ZNF92* have high homology with *ZNF117* and cluster on human chromosome 7: "The physiological function of *ZNF117* has not been clarified, but it seems possible that this gene contributes to the biological effects of *ERV3*" (or vice versa !).

*ZNF117* encodes for Zinc Finger Protein 117 (Uniprot: Q03924), almost certainly a transcription factor. Three *ZNF117* transcripts are presented in Ensembl (Fig. 3;^38^): ENST00000282869.11 = *ZNF117*-201 and ENST00000620222.4 = *ZNF117*-203 code for a 483 amino-acid protein; ENST00000487644.1 = *ZNF117*-202 has no protein. *ZNF117*-202 has two interesting features: (1) it has an additional exon upstream; and (2) rs67047829 is found in the retained intron, ~ 2 kbp (base-pairs) upstream from *ZNF117*-201 and *ZNF117*-203 (source: dbSNP; *ZNF117*-202 has tag TSL-2 = intermediate evidential support). (*ZNF117* also has its own pretermination codon, rs1404453, which was not possible to study here, and which does not have high linkage disequilibrium with rs67047829: the truncated version is more prevalent than the non-truncated version.)

It is well known that regulatory elements can act from upstream code, and recently it has even been suggested that alternative start codons exist for ~ 12,000 potential regulatory upstream open reading frames^39^. As from *ZNF117*-202 we already know that an alternative upstream exon is a possibility, perhaps this applies to *ZNF117* protein-coding transcripts ? In any case, we can speculate that rs67047829 (or a linked region) lies within the regulatory region of some *ZNF117* protein-coding transcripts. Bustamante-Rivera et al.^40^ analyzed the untranslated regions of read-through transcripts and suggested these might have fewer binding sites for microRNAs and non-coding RNAs and that "whether the different *ZNF117* transcripts have different stabilities and translation efficiencies should be analyzed."

The Zinc Finger Protein 117 itself has been suggested to affect subcutaneous obesity and visceral or abdominal-visceral obesity^41^ in a study of adipose stem cells and, in a study using preadipocyte single-nuclei RNA sequencing, this was one of the transcription factors found with activity exclusively in brown adipogenesis (and therefore presumably linked to thermogenic response)^42^. Additionally, *ZNF117* and *ONECUT2* gave the only two transcription factors upregulated in expression-quantitative-trait loci analysis from laser-capture-microdissected pancreatic islet cells from patients with type-2 diabetes^43^.

There is now, therefore, evidence that *ZNF117* is implicated in a cellular preponderance for obesity-related effects, and from the association in the present study it is at least possible that rs67047829 itself, or a linked region, might change the upstream regulatory region for this gene (or, alternatively, *ERV3-1* mRNA with rs67047829 might somehow affect *ZNF117* mRNA processing as they share a common exonic region, in ways that are not fully understood).

### *ERV3-1*

A second hypothesis, with rs67047829 acting as a pretermination codon in *ERV3-1* to affect BMI, is perhaps a simpler hypothesis. The protein expressed from *ERV3-1* is the Endogenous Retrovirus Group 3 Member 1 Env Polyprotein (UNIPROT Q14264; here referred to as ERV3-1env). The rs67047829:A allele provides a pretermination codon in the SU domain probably, but not necessarily, resulting in mRNA degradation before a protein can be produced. If ERV3-1env somehow stimulates adipose-cell proliferation, then removal via this PTC-SNP might confer protective effects or alternatively a truncated protein, if expressed, might confer these effects.

Although most HERV remnants are virally inactive, protein expression still often occurs and HERVs are associated with several autoimmune diseases^44^. ERV3-1env has not been found to compose viral particles and it has lost its fusogenic properties^45^. However, as its open reading frame has been conserved through 30 million years of primate evolution, and as full-length proteins (from four exons) are expressed in many tissues^46^, it likely has biological functions beneficial to the host^47^. In a placental trophoblast model with BeWo cells stably transfected with ERV3-1env, beta human chorionic gonadotrophin expression, which positively regulates the cell cycle^48^, was increased; cyclin B expression, which promotes cell cycling, was reduced; while p21 expression, which negatively regulates the cell cycle, was up-regulated^49^.

ERV3-1env is composed of two major domains, SU and TM (which split from each other during processing and are then held together by non-covalent bonds). In a virus, SU would mediate receptor recognition whereas TM would be a transmembrane domain. If *ERV3-1* with the rs67047829:A allele did produce a protein this would be without the TM domain with 222 rather than 604 amino acids.

Of possible direct relevance to the result found here is that, although ERV3-1env mRNA is found in all tissues, it is highly expressed in adipose tissue. According to the Human Protein Atlas (https://www.proteinatlas.org^46^), adipose tissue had the highest *ERV3-1* mRNA expression in one database (number of tissues: 45) and had within the top four mRNA expression levels in the other databases. Note that, conversely, low protein expression was found in adipose tissue.

For *ERV-3-1*-201 the type of NMD activated cannot follow the 50 nucleotide intron rule^50,51^ as there are no introns after the transcribed region containing the protein coding region, with or without the PTC (Fig. 3), but NMD occurs via many different mechanisms and intron-less mRNAs are regularly degraded^52^. Of interest is the fact that NMD degrades some but not all mRNAs bearing PTCs^53^ and many PTC-containing transcripts are likely to be translated into truncated and/or partially-frameshifted proteins^53^. Lack of expression of a truncated ERV3-1env protein should not therefore be assumed and if it escapes NMD degradation this might result in high protein expression in adipose tissue from the known high mRNA expression. Details of protein biological function for the reference protein or the truncated version are, unfortunately, not known.

**Figure 4:**
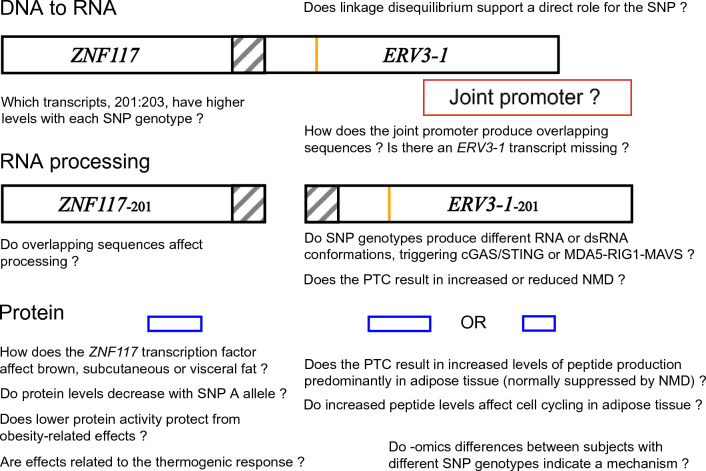
Diagrammatic representation of *ERV3-1/ZNF117* expression. Questions concerning *ERV3-1/ZNF117* expression, the exonic region overlap (striped boxes), the single nucleotide polymorphism (SNP) rs67047829 (orange lines), and proteins produced (blue boxes). cGAS/STING and MDA5-RIG1-MAVS are metabolic pathways (see “Discussion”); *NMD* nonsense-mediated RNA decay, *PTC* pretermination codon, *dsRNA* double-stranded RNA.

Another possibility is that conformational change in RNA or double-stranded RNA caused by rs67047829 might trigger the cGAS/STING or MDA5-RIG1-MAVS pathways (Fig. 4). cGAS/STING is the cyclic GMP–AMP synthase (cGAS) stimulator of interferon genes (STING) pathway; and MDA5-RIG1-MAVS results from MDA5 and RIG-I as cytoplasmic viral RNA sensors with MAVS is their common signaling adaptor molecule. Either of these might affect innate immunity and perhaps inhibit thermogenic gene expression or contribute to obesity induced by overnutrition^54^.

### Effects from both *ERV3-1 *and *ZNF117*?

Expression of *ERV3-1* and *ZNF117* might be linked in complex ways. According to Bustamante-Rivera^40^ the "*ERV3* locus can be considered as an alternative promoter for *ZNF117*", and the present annotation indicates a shared promoter region (Fig. 3). Perhaps, at least sometimes, the *ERV3-1*-derived long noncoding RNA (lncRNA; named TROJAN, Stricker et al.^55^) forms a first nuclear RNA transcript which is then, in healthy cells, spliced into various transcripts of both *ERV3-1* and *ZNF117*. (Other potential *ERV*/zinc-finger read-throughs are known e.g. *ERV-ZNF8*^40^).

It should also be noted that at least one transcript each of *ERV3-1* and *ZNF117* share a common untranslated exon, which means that if there are mechanisms which target this region then both genes will be affected. It is also unclear whether the methods used to measure *ERV3-1* mRNA transcripts are sufficiently accurate to distinguish from *ZNF117* mRNA transcripts, in which case perhaps the latter also has high expression in adipose tissue (?).

As the detailed biological functions of both proteins are at present also unclear (the protein from *ERV3-1* is somehow involved in cell cycling, and that from *ZNF117* is almost certainly a transcription factor), some of these questions will only be answered after detailed protein biological functions (or RNA or dsRNA effects) have been elucidated (see Fig. 4). The simplest hypothesis seems to be that the functional effect of the pretermination codon on *ERV3-1* is associated with obesity, but effects via *ZNF117* cannot be dismissed.

Therefore, although evidence is provided here that rs67047829 or a linked region might be associated with lower BMI, if this is confirmed then considerable further work will still be required to determine if a causal effect exists and whether this is related to ERV3-1env truncation or whether this results from differential expression from either the *ERV3-1* or the *ZNF117* gene, or both. Additionally, investigation of linkage disequilibrium around rs67047829 and -omics studies comparing subjects with each genotype might elucidate a role for the *ERV3-1/ZNF117* locus in obesity.

## Conclusions

In conclusion, datamining of high-frequency pretermination-codon single nucleotide polymorphisms, in data from a large sample of the Polish population (n = 5757), has resulted in the discovery of an association between rs67047829 and BMI in otherwise healthy subjects. rs67047829 forms a pretermination codon in *ERV3-1* and potentially lies in a regulatory region of *ZNF117*, which has various intriguing connections with *ERV3-1* and is already known to have association with cellular adipogenesis and type-2 diabetes. If causal, this might indicate a large protective effect of the rs67047829:A allele or a linked region against BMI increase. A drug targeting *ZNF117* or *ERV3-1* regulation might well not have dangerous side-effects as this result was found in a Mendelian setting with near-neutral selection. Further study involving an even larger cohort or from another (e.g. Asian) ethnic group is needed to confirm this result and then functional studies are also needed to decipher potential effects.

### Supplementary Information

Supplementary Table 1.

Supplementary Table 2.

Supplementary Table 3.

Supplementary Information 4.

Supplementary Information 5.

## Data Availability

All coding for all results, and all data, are found in Supplementary Information here and also at https://github.com/Abiologist/PTCobesity.git (to download file from github: right-click on "raw", "Save Link As" might be necessary).
