# Supplementary material for: rs67047829 genotypes of ERV3-1/ZNF117 are associated with lower body mass index in the Polish population
Source: Sci Rep. 2023 Oct 10;13:17118. doi: 10.1038/s41598-023-43323-3 (PMC10564729; doi:10.1038/s41598-023-43323-3)
Supplement: Supplementary file 4 — Supplementary Information 4. [file 41598_2023_43323_MOESM4_ESM.docx]

## Supplementary_file_S4.Coding for all results (except power study; see Supplementary_file_S5): including datamining results and final regressions for males and females separately and together, plus calculations for Table 1 and odds ratios.
## Article: "rs67047829 genotypes of ERV3-1/ZNF117 are associated with lower body mass index in the Polish population."
## Authors: Jeremy Clark*, Konrad Podsiadło*, Marta Sobalska-Kwapis, Błażej Marciniak, Kamila Rydzewska, Andrzej Ciechanowicz, Thierry van de Wetering, Dominik Strapagiel

## INSTRUCTIONS: 1. Create .csv files from .xlsx Supplementary files by removing all header rows bar one from Supplementary_Table_S2 and Supplementary_Table_S3. 2. Run code between lines below: make sure all libraries are TRUE and "mypath" to files correctly gives mydf2. Copy and paste all coding into R. (This coding has been run on Windows 11 Pro and Mac 10.15.7).

##_________________________________________________________________
ipak <- function(pkg){
new.pkg <- pkg[!(pkg %in% installed.packages()[, "Package"])]
if (length(new.pkg))
 install.packages(new.pkg, dependencies = TRUE, repos="http://cran.r-project.org")
sapply(pkg, require, character.only = TRUE)
} ## end function
packages <- c("data.table", "car", "dplyr", "SNPassoc", "rlist", "epitools")
options(timeout=1000)
ipak(packages)

mypath <- "/Users/jeremyclark/Documents/aaJ Clark MACBOOK 2022 PUM/3 AA SUBMITTED PTCobesity Scientific Reports 140 pt/FIGS AND SUPP/"
mydf2 <- fread(paste0(mypath, "Supplementary_Table_S2_D.csv"))
head(mydf2)
nrow(mydf2)
mydf2 <- as.data.frame(mydf2)
colnames(mydf2) <- make.names(colnames(mydf2), unique = TRUE)
colnames(mydf2)

##_________________________________________________________________

## Change n.d. to NA throughout dataframe:
mydf2[mydf2 == "n.d."] <- NA

levels(as.factor(mydf2[ , "Age"]))
mydf2[ , "Age"] <- as.numeric(mydf2[ , "Age"])
sum(is.na(mydf2[ , "Age"]))
levels(as.factor(mydf2[ , "BMI"]))
mydf2[ , "BMI"] <- as.numeric(mydf2[ , "BMI"])
sum(is.na(mydf2[ , "BMI"]))
levels(as.factor(mydf2[ , "SEX"]))
sum(is.na(mydf2[ , "SEX"]))

nrow(mydf2)

## To obtain all numbers found in Supplementary_Table_S3:

## only those subjects analyzed with data for Age, BMI and SEX, n = 5050:

mydfA <- mydf2[complete.cases(mydf2[ , c("Age", "BMI", "SEX")]), ]
head(mydfA)
colnames(mydfA)
nrow(mydfA)

## remove all duplicate columns with _B:
dfNoDup <- mydfA %>% select(-contains("_B"))
colnames(dfNoDup)
dfGENnoPHEN <- dfNoDup[ , 11:ncol(dfNoDup)]
names(dfGENnoPHEN) <- gsub("_A", "", names(dfGENnoPHEN), fixed = TRUE)
colnames(dfGENnoPHEN)

## create genotype column dataframe:
dfgen_1 <- dfGENnoPHEN[ , c(TRUE, FALSE)]
colnames(dfgen_1)
ncol(dfgen_1)
head(dfgen_1)
dfgen_2 <- dfGENnoPHEN[ , c(FALSE, TRUE)]
colnames(dfgen_2)
ncol(dfgen_2)
head(dfgen_2)
mycol <- list();
for (i in 1:ncol(dfgen_1)) {
mycol[[i]] <- paste0(dfgen_1[ , i], "/", dfgen_2[ , i])
names(mycol)[i] <- colnames(dfgen_1)[i]
}
head(mycol[[1]])
GENdf3 <- list.cbind(mycol)
head(GENdf3)
GENdf2 <- GENdf3
for (i in 1:ncol(GENdf3)) {
GENdf2[ , i] <- gsub("NA/NA", "NA", GENdf3[ , i])
}
head(GENdf2)
## add phenotype back:
GENdf <- cbind(mydfA[ , 1:10], GENdf2)
head(GENdf)
colnames(GENdf)

for (i in 11:ncol(GENdf)) {
GENdf[ , i] <- as.factor(GENdf[ , i])
}

ANALYSIS <- setupSNP(GENdf, colSNPs = c(11:ncol(GENdf)), sep = "/")

TABLE_BMI3 <- WGassociation(BMI ~ Age + SEX, model = c("dominant", "recessive", "overdominant", "log-additive"), data = ANALYSIS, genotypingRate = 0.1)
TABLE_BMI3 <- as.data.frame(TABLE_BMI3)
TABLE_BMI3$rs_number <- rownames(TABLE_BMI3)
TABLE_BMI2 <- TABLE_BMI3 %>% dplyr::select("rs_number", everything())

## rearrange order of SNPs to match those in Supplementary_Table_3:
Table_S3 <- fread(paste0(mypath, "Supplementary_Table_S3_J.csv"))

nrow(Table_S3)
Table_S3 <- as.data.frame(Table_S3)
colnames(Table_S3)
colnames(Table_S3)[2] <- "rs_number"
colnames(Table_S3)

rs_S3 <- Table_S3[ , "rs_number"]
TABLE_BMI <- TABLE_BMI2[match(rs_S3, TABLE_BMI2$rs_number), ]
TABLE_BMI <- TABLE_BMI[1:139, ]
TABLE_BMI

for (i in 3:ncol(TABLE_BMI)) {
for (j in 1:nrow(TABLE_BMI)) {
TABLE_BMI[j, i] <- signif(TABLE_BMI[j, i], 3)
}
}

## write .csv file:
write.csv(TABLE_BMI, paste0(mypath, "TABLE_BMI.csv"))

## False discovery rate correction:
dominant <- TABLE_BMI[ , "dominant"]
recessive <- TABLE_BMI[ , "recessive"]
overdominant <- TABLE_BMI[ , "overdominant"]
logadditive <- TABLE_BMI[ , "log-additive"]

pvaluevec <- c(dominant, recessive, overdominant, logadditive)
padjustvec <- p.adjust(pvaluevec, method = c("BY"), n = length(pvaluevec))
padjustvec

## rs67047829 association with body mass Index. subjects with >5% missing SNPs removed. n = 4425
names(mydf2) <- gsub("rs67047829", "rs67047829_1", names(mydf2), fixed = TRUE)
names(mydf2) <- gsub("rs67047829_1.1", "rs67047829_2", names(mydf2), fixed = TRUE)
colnames(mydf2)

rs67047829 <- paste0(mydf2[ , "rs67047829_1"], mydf2[ , "rs67047829_2"])
mydf2$rs67047829 <- rs67047829
levels(as.factor(mydf2[ , "rs67047829"]))
sum(is.na(mydf2[ , "rs67047829"]))

## Counts of rs67047829 genotypes - mydf2:
levels(as.factor(mydf2[ , "rs67047829"]))
Genomydf2 <- mydf2[ , "rs67047829"]
Genomydf2_AA <- Genomydf2[Genomydf2 == "AA"]
head(Genomydf2_AA)
length(Genomydf2_AA)
Genomydf2_GG <- Genomydf2[Genomydf2 == "GG"]
head(Genomydf2_GG)
length(Genomydf2_GG)
Genomydf2_GA <- Genomydf2[Genomydf2 == "GA"]
head(Genomydf2_GA)
length(Genomydf2_GA)
Genomydf2_AG <- Genomydf2[Genomydf2 == "AG"]
head(Genomydf2_AG)
length(Genomydf2_AG)

## Remove subject if data missing from Age, BMI, SEX or rs67047829:

mydf1B <- mydf2[complete.cases(mydf2[ , c("Age", "BMI")]), ]
head(mydf1B)
nrow(mydf1B)
colnames(mydf1B)

mydf1C <- mydf2[complete.cases(mydf2[ , c("SEX", "BMI")]), ]
head(mydf1C)
colnames(mydf1C)
nrow(mydf1C)

mydf1D <- mydf2[complete.cases(mydf2[ , c("BMI")]), ]
head(mydf1D)
colnames(mydf1D)
nrow(mydf1D)

mydf1 <- mydf2[complete.cases(mydf2[ , c("Age", "BMI", "SEX", "rs67047829")]), ]
head(mydf1)
nrow(mydf1)
colnames(mydf1)

## Counts of genotypes - mydf1
levels(as.factor(mydf1[ , "rs67047829"]))
Genomydf1 <- mydf1[ , "rs67047829"]
Genomydf1_AA <- Genomydf1[Genomydf1 == "AA"]
head(Genomydf1_AA)
length(Genomydf1_AA)
Genomydf1_GG <- Genomydf1[Genomydf1 == "GG"]
head(Genomydf1_GG)
length(Genomydf1_GG)
Genomydf1_GA <- Genomydf1[Genomydf1 == "GA"]
head(Genomydf1_GA)
length(Genomydf1_GA)
Genomydf1_AG <- Genomydf1[Genomydf1 == "AG"]
head(Genomydf1_AG)
length(Genomydf1_AG)

sum(is.na(mydf1[ , "Age"]))
sum(is.na(mydf1[ , "BMI"]))
sum(is.na(mydf1[ , "SEX"]))
sum(is.na(mydf1[ , "rs67047829"]))

## Find subjects with more than a certain percentage of genotype fails:
NoPhendf1 <- mydf1[ , 11:ncol(mydf1)]
head(NoPhendf1)
ncol(NoPhendf1)
nrow(NoPhendf1)

NAs <- rowSums(is.na(NoPhendf1))
max(NAs)

## <8 missing gives <5% missing SNP values n = 4425
## <22 missing gives <15% missing SNP values n = 5026

NAtrue <- list();
for (i in 1:length(NAs)) {
if (NAs[[i]] < 8) { ## <8 missing gives <5% missing SNP values n = 4425
NAtrue[[i]] <- TRUE
} else {
NAtrue[[i]] <- FALSE
}
}
NAtrue <- as.vector(unlist(NAtrue))

mydf <- mydf1[NAtrue, ]
nrow(mydf)
ncol(mydf)

mydf[ , "BMI"] <- as.numeric(mydf[ , "BMI"])
mydf[ , "Age"] <- as.numeric(mydf[ , "Age"])

## Genotype <- paste0(mydf[ , "rs67047829_1"], mydf[ , "rs67047829_2"])

levels(as.factor(mydf[ , "rs67047829"]))
Genomydf <- mydf[ , "rs67047829"]
Genomydf_AA <- Genomydf[Genomydf == "AA"]
head(Genomydf_AA)
length(Genomydf_AA)
Genomydf_GG <- Genomydf[Genomydf == "GG"]
head(Genomydf_GG)
length(Genomydf_GG)
Genomydf_GA <- Genomydf[Genomydf == "GA"]
head(Genomydf_GA)
length(Genomydf_GA)
Genomydf_AG <- Genomydf[Genomydf == "AG"]
head(Genomydf_AG)
length(Genomydf_AG)


Genotype <- mydf[ , "rs67047829"] ## Genotype given as e.g. "GG"

levels(as.factor(Genotype))

mydf$Genotype <- Genotype
head(mydf)
## Dominant model coding:
Dominant <- Genotype
Dominant[(Genotype == "GA") | (Genotype == "AG") | (Genotype == "AA")] <- 1
Dominant[Genotype == "GG"] <- 0
mydf$Dominant <- as.factor(Dominant)
## Recessive model coding:
Recessive <- Genotype
Recessive[Genotype == "AA"] <- 0
Recessive[(Genotype == "GA") | (Genotype == "AG") | (Genotype == "GG")] <- 1
mydf$Recessive <- as.factor(Recessive)
## Heterozygote model coding:
Heterozygote <- Genotype
Heterozygote[(Genotype == "GA") | (Genotype == "AG")] <- 1
Heterozygote[(Genotype == "GG") | (Genotype == "AA")] <- 0
mydf$Heterozygote <- as.factor(Heterozygote)
## Additive model coding:
Additive <- Genotype
Additive[Genotype == "GG"] <- 0
Additive[(Genotype == "GA") | (Genotype == "AG")] <- 1
Additive[Genotype == "AA"] <- 2

mydf$Additive <- as.factor(Additive)

levels(as.factor(Genotype))
levels(as.factor(mydf[ , "BMI"]))
levels(as.factor(mydf[ , "SEX"]))
levels(as.factor(mydf[ , "Age"]))
levels(as.factor(Dominant))
levels(as.factor(Recessive))
levels(as.factor(Heterozygote))
levels(as.factor(Additive))

AA <- Genotype[Genotype == "AA"]
AA
AG <- Genotype[Genotype == "AG"]
AG
GA <- Genotype[Genotype == "GA"]
GA
GG <- Genotype[Genotype == "GG"]
GG


## BMI vs. recessive logistic model, adjusted for year of birth and SEX.

summary(glm(mydf$Recessive ~ mydf$BMI + mydf$Age + mydf$SEX, family = binomial, data = mydf))

## Call:
## glm(formula = mydf$Recessive ~ mydf$BMI + mydf$Age + mydf$SEX,
## family = binomial, data = mydf)
##
## Deviance Residuals:
## Min 1Q Median 3Q Max
## -3.2251 0.1127 0.1377 0.1601 0.2675
##
## Coefficients:
## Estimate Std. Error z value Pr(>|z|)
## (Intercept) 2.060997 0.998425 2.064 0.03899 *
## mydf$BMI 0.130099 0.044079 2.951 0.00316 **
## mydf$Age -0.008356 0.010768 -0.776 0.43775
## mydf$SEX -0.183445 0.312723 -0.587 0.55747
## ---
## Signif. codes: 0 ‘***’ 0.001 ‘**’ 0.01 ‘*’ 0.05 ‘.’ 0.1 ‘ ’ 1
##
## (Dispersion parameter for binomial family taken to be 1)
##
## Null deviance: 493.31 on 4424 degrees of freedom
## Residual deviance: 483.38 on 4421 degrees of freedom
## AIC: 491.38
##
## Number of Fisher Scoring iterations: 8

## Result: low p value for BMI, and main effects of age and sex are not significant - and these adjustors are now removed:

## BMI vs. recessive logistic model, adjustments removed.

Rec <- glm(mydf$Recessive ~ mydf$BMI, family = binomial, data = mydf)
summary(Rec)

## Call:
## glm(formula = mydf$Recessive ~ mydf$BMI, family = binomial, data = mydf)
##
## Deviance Residuals:
## Min 1Q Median 3Q Max
## -3.2559 0.1147 0.1372 0.1598 0.2526
##
## Coefficients:
## Estimate Std. Error z value Pr(>|z|)
## (Intercept) 1.83481 0.94785 1.936 0.05290 .
## mydf$BMI 0.11377 0.03995 2.847 0.00441 **
## ---
## Signif. codes: 0 ‘***’ 0.001 ‘**’ 0.01 ‘*’ 0.05 ‘.’ 0.1 ‘ ’ 1
##
## (Dispersion parameter for binomial family taken to be 1)
##
## Null deviance: 493.31 on 4424 degrees of freedom
## Residual deviance: 484.27 on 4423 degrees of freedom
## AIC: 488.27
##
## Number of Fisher Scoring iterations: 8

## BETA: Recessive
Recmodel <- Rec$model
Recmodel <- as.data.frame(sapply(Recmodel, as.numeric))
RecScale1 <- data.frame(scale(Recmodel[ , 2]))
RecScale <- cbind(Recmodel[ , 1], RecScale1)
colnames(RecScale) <- c("Recessive", "BMIScale")
RecScale$Recessive <- as.factor(RecScale$Recessive)
levels(RecScale$Recessive) <- list("0" = "1", "1" = "2")
RecScale.glm <- glm(RecScale$Recessive ~ RecScale$BMI, family = binomial, data = RecScale)
summary(RecScale.glm)

## Call:
## glm(formula = RecScale$Recessive ~ RecScale$BMI, family = binomial,
## data = RecScale)
##
## Deviance Residuals:
## Min 1Q Median 3Q Max
## -3.2559 0.1147 0.1372 0.1598 0.2526
##
## Coefficients:
## Estimate Std. Error z value Pr(>|z|)
## (Intercept) 4.7155 0.1697 27.793 < 2e-16 ***
## RecScale$BMI 0.5159 0.1812 2.847 0.00441 **
## ---
## Signif. codes: 0 ‘***’ 0.001 ‘**’ 0.01 ‘*’ 0.05 ‘.’ 0.1 ‘ ’ 1
##
## (Dispersion parameter for binomial family taken to be 1)
##
## Null deviance: 493.31 on 4424 degrees of freedom
## Residual deviance: 484.27 on 4423 degrees of freedom
## AIC: 488.27
##
## Number of Fisher Scoring iterations: 8


confint(RecScale.glm)
## 2.5 % 97.5 %
## (Intercept) 4.4027713 5.071126
## RecScale$BMI 0.1729828 0.883053

## Result: Final low p value for BMI with the recessive model.


## BMI vs. dominant model, adjusted for year of birth, SEX.

summary(glm(mydf$Dominant ~ mydf$BMI + mydf$Age + mydf$SEX, family = binomial, data = mydf))

## Call:
## glm(formula = mydf$Dominant ~ mydf$BMI + mydf$Age + mydf$SEX,
## family = binomial, data = mydf)
##
## Deviance Residuals:
## Min 1Q Median 3Q Max
## -0.7134 -0.6385 -0.6136 -0.5739 2.0571
##
## Coefficients:
## Estimate Std. Error z value Pr(>|z|)
## (Intercept) -0.900294 0.247965 -3.631 0.000283 ***
## mydf$BMI -0.027219 0.009760 -2.789 0.005291 **
## mydf$Age 0.001324 0.002848 0.465 0.641995
## mydf$SEX -0.013993 0.080625 -0.174 0.862210
## ---
## Signif. codes: 0 ‘***’ 0.001 ‘**’ 0.01 ‘*’ 0.05 ‘.’ 0.1 ‘ ’ 1
##
## (Dispersion parameter for binomial family taken to be 1)
##
## Null deviance: 4105.9 on 4424 degrees of freedom
## Residual deviance: 4097.2 on 4421 degrees of freedom
## AIC: 4105.2
##
## Number of Fisher Scoring iterations: 4


## Result: BMI p-value for Dominant model highly signficant, almost as low as recessive model with adjustors. Main effects of Age and SEX are not significant and these adjustors are removed below:

## BMI vs. dominant model, adjustors removed.

Dom <- glm(mydf$Dominant ~ mydf$BMI, family = binomial, data = mydf)
summary(Dom)

## Call:
## glm(formula = mydf$Dominant ~ mydf$BMI, family = binomial, data = mydf)
##
## Deviance Residuals:
## Min 1Q Median 3Q Max
## -0.7067 -0.6382 -0.6141 -0.5744 2.0625
##
## Coefficients:
## Estimate Std. Error z value Pr(>|z|)
## (Intercept) -0.895394 0.229694 -3.898 9.69e-05 ***
## mydf$BMI -0.026014 0.009058 -2.872 0.00408 **
## ---
## Signif. codes: 0 ‘***’ 0.001 ‘**’ 0.01 ‘*’ 0.05 ‘.’ 0.1 ‘ ’ 1
##
## (Dispersion parameter for binomial family taken to be 1)
##
## Null deviance: 4105.9 on 4424 degrees of freedom
## Residual deviance: 4097.5 on 4423 degrees of freedom
## AIC: 4101.5
##
## Number of Fisher Scoring iterations: 4

## BETA: Dominant
Dommodel <- Dom$model
Dommodel <- as.data.frame(sapply(Dommodel, as.numeric))
DomScale1 <- data.frame(scale(Dommodel[ , 2]))
DomScale <- cbind(Dommodel[ , 1], DomScale1)
colnames(DomScale) <- c("Dominant", "BMIScale")
DomScale$Dominant <- as.factor(DomScale$Dominant)
levels(DomScale$Dominant) <- list("0" = "1", "1" = "2")

DomScale.glm <- glm(DomScale$Dominant ~ DomScale$BMI, family = binomial, data = DomScale)
summary(DomScale.glm)

## Call:
## glm(formula = DomScale$Dominant ~ DomScale$BMI, family = binomial,
## data = DomScale)
##
## Deviance Residuals:
## Min 1Q Median 3Q Max
## -0.7067 -0.6382 -0.6141 -0.5744 2.0625
##
## Coefficients:
## Estimate Std. Error z value Pr(>|z|)
## (Intercept) -1.55409 0.03971 -39.137 < 2e-16 ***
## DomScale$BMI -0.11797 0.04108 -2.872 0.00408 **
## ---
## Signif. codes: 0 ‘***’ 0.001 ‘**’ 0.01 ‘*’ 0.05 ‘.’ 0.1 ‘ ’ 1
##
## (Dispersion parameter for binomial family taken to be 1)
##
## Null deviance: 4105.9 on 4424 degrees of freedom
## Residual deviance: 4097.5 on 4423 degrees of freedom
## AIC: 4101.5
##
## Number of Fisher Scoring iterations: 4


confint(DomScale.glm)
## 2.5 % 97.5 %
## (Intercept) -1.6325957 -1.47691670
## DomScale$BMI -0.1991985 -0.03816629

## Result: Final low p value for dominant model is lower than for recessive model.


## BMI vs. heterozygote model, adjusted for year of birth, SEX.

summary(glm(mydf$Heterozygote ~ mydf$BMI + mydf$Age + mydf$SEX, family = binomial, data = mydf))

## Call:
## glm(formula = mydf$Heterozygote ~ mydf$BMI + mydf$Age + mydf$SEX,
## family = binomial, data = mydf)
##
## Deviance Residuals:
## Min 1Q Median 3Q Max
## -0.6700 -0.6147 -0.5958 -0.5678 2.0462
##
## Coefficients:
## Estimate Std. Error z value Pr(>|z|)
## (Intercept) -1.1010704 0.2529082 -4.354 1.34e-05 ***
## mydf$BMI -0.0205800 0.0098913 -2.081 0.0375 *
## mydf$Age 0.0008446 0.0029148 0.290 0.7720
## mydf$SEX -0.0245934 0.0824448 -0.298 0.7655
## ---
## Signif. codes: 0 ‘***’ 0.001 ‘**’ 0.01 ‘*’ 0.05 ‘.’ 0.1 ‘ ’ 1
##
## (Dispersion parameter for binomial family taken to be 1)
##
## Null deviance: 3966.5 on 4424 degrees of freedom
## Residual deviance: 3961.4 on 4421 degrees of freedom
## AIC: 3969.4
##
## Number of Fisher Scoring iterations: 4

## Result: Heterozygote model significant with adjustments for Age and SEX - adjustor main effects are not significant - and can be removed:


## BMI vs. heterozygote model, adjustors removed.

Het <- glm(mydf$Heterozygote ~ mydf$BMI, family = binomial, data = mydf)
summary(Het)

## Call:
## glm(formula = mydf$Heterozygote ~ mydf$BMI, family = binomial,
## data = mydf)
##
## Deviance Residuals:
## Min 1Q Median 3Q Max
## -0.6653 -0.6149 -0.5965 -0.5682 2.0485
##
## Coefficients:
## Estimate Std. Error z value Pr(>|z|)
## (Intercept) -1.113745 0.233854 -4.763 1.91e-06 ***
## mydf$BMI -0.020104 0.009195 -2.186 0.0288 *
## ---
## Signif. codes: 0 ‘***’ 0.001 ‘**’ 0.01 ‘*’ 0.05 ‘.’ 0.1 ‘ ’ 1
##
## (Dispersion parameter for binomial family taken to be 1)
##
## Null deviance: 3966.5 on 4424 degrees of freedom
## Residual deviance: 3961.6 on 4423 degrees of freedom
## AIC: 3965.6
##
## Number of Fisher Scoring iterations: 4

## BETA: Heterozygote
Hetmodel <- Het$model
Hetmodel <- as.data.frame(sapply(Hetmodel, as.numeric))
HetScale1 <- data.frame(scale(Hetmodel[ , 2]))
HetScale <- cbind(Hetmodel[ , 1], HetScale1)
colnames(HetScale) <- c("Heterozygote", "BMIScale")
HetScale$Heterozygote <- as.factor(HetScale$Heterozygote)
levels(HetScale$Heterozygote) <- list("0" = "1", "1" = "2")

HetScale.glm <- glm(HetScale$Heterozygote ~ HetScale$BMI, family = binomial, data = HetScale)
summary(HetScale.glm)

## Call:
## glm(formula = HetScale$Heterozygote ~ HetScale$BMI, family = binomial,
## data = HetScale)
##
## Deviance Residuals:
## Min 1Q Median 3Q Max
## -0.6653 -0.6149 -0.5965 -0.5682 2.0485
##
## Coefficients:
## Estimate Std. Error z value Pr(>|z|)
## (Intercept) -1.62281 0.04058 -39.990 <2e-16 ***
## HetScale$BMI -0.09117 0.04170 -2.186 0.0288 *
## ---
## Signif. codes: 0 ‘***’ 0.001 ‘**’ 0.01 ‘*’ 0.05 ‘.’ 0.1 ‘ ’ 1
##
## (Dispersion parameter for binomial family taken to be 1)
##
## Null deviance: 3966.5 on 4424 degrees of freedom
## Residual deviance: 3961.6 on 4423 degrees of freedom
## AIC: 3965.6
##
## Number of Fisher Scoring iterations: 4

confint(HetScale.glm)
## 2.5 % 97.5 %
## (Intercept) -1.7030699 -1.54397314
## HetScale$BMI -0.1736479 -0.01017095

## Result: Heterozygote model is significant.


## BMI vs. additive model, adjusted for year of birth, SEX - linear regression.

Additive <- as.factor(Additive)

## I The coefficient of determination (r 2 ) of an additive linear regression model gives an estimate of the proportion of phenotypic variation that is explained by the SNP (or SNPs) in the model, e.g., the ”SNP heritability”
## http://faculty.washington.edu/tathornt/SISG2017/lectures/SISG2017session02.pdf

Anova(lm(mydf$BMI ~ mydf$Additive + mydf$Age + mydf$SEX), type = "II", white.adjust = TRUE)

## Coefficient covariances computed by hccm()
## Analysis of Deviance Table (Type II tests)
##
## Response: mydf$BMI
## Df F Pr(>F)
## mydf$Additive 2 10.131 4.076e-05 ***
## mydf$Age 1 543.209 < 2.2e-16 ***
## mydf$SEX 1 126.717 < 2.2e-16 ***
## Residuals 4420
## ---
## Signif. codes: 0 ‘***’ 0.001 ‘**’ 0.01 ‘*’ 0.05 ‘.’ 0.1 ‘ ’ 1

## Result: additive model is significant - but Age and Sex main effects are also very significant - therefore assess interactions:

Anova(lm(mydf$BMI ~ mydf$Additive * mydf$Age * mydf$SEX), type = "II", white.adjust = TRUE)

## Coefficient covariances computed by hccm()
## Analysis of Deviance Table (Type II tests)
##
## Response: mydf$BMI
## Df F Pr(>F)
## mydf$Additive 2 10.1872 3.854e-05 ***
## mydf$Age 1 518.6202 < 2.2e-16 ***
## mydf$SEX 1 161.0734 < 2.2e-16 ***
## mydf$Additive:mydf$Age 2 0.2551 0.7749
## mydf$Additive:mydf$SEX 2 0.5169 0.5964
## mydf$Age:mydf$SEX 1 58.9407 1.988e-14 ***
## mydf$Additive:mydf$Age:mydf$SEX 2 2.0019 0.1352
## Residuals 4413
## ---
## Signif. codes: 0 ‘***’ 0.001 ‘**’ 0.01 ‘*’ 0.05 ‘.’ 0.1 ‘ ’ 1

## Result: Age and Sex interactions with BMI are not significant - therefore remove interactions, but not adjustors

Anova(lm(mydf$BMI ~ mydf$Additive + mydf$Age + mydf$SEX), type = "II", white.adjust = TRUE)

## Coefficient covariances computed by hccm()
## Analysis of Deviance Table (Type II tests)
##
## Response: mydf$BMI
## Df F Pr(>F)
## mydf$Additive 2 10.131 4.076e-05 ***
## mydf$Age 1 543.209 < 2.2e-16 ***
## mydf$SEX 1 126.717 < 2.2e-16 ***
## Residuals 4420
## ---
## Signif. codes: 0 ‘***’ 0.001 ‘**’ 0.01 ‘*’ 0.05 ‘.’ 0.1 ‘ ’ 1

Add <- lm(mydf$BMI ~ mydf$Additive + mydf$Age + mydf$SEX)
summary(Add)

## Call:
## lm(formula = mydf$BMI ~ mydf$Additive + mydf$Age + mydf$SEX)
##
## Residuals:
## Min 1Q Median 3Q Max
## -11.508 -2.870 -0.556 2.363 36.397
##
## Coefficients:
## Estimate Std. Error t value Pr(>|t|)
## (Intercept) 19.013083 0.274758 69.199 < 2e-16 ***
## mydf$Additive1 -0.379329 0.171163 -2.216 0.02673 *
## mydf$Additive2 -1.920195 0.640589 -2.998 0.00274 **
## mydf$Age 0.101075 0.004305 23.480 < 2e-16 ***
## mydf$SEX 1.424091 0.127107 11.204 < 2e-16 ***
## ---
## Signif. codes: 0 ‘***’ 0.001 ‘**’ 0.01 ‘*’ 0.05 ‘.’ 0.1 ‘ ’ 1
##
## Residual standard error: 4.224 on 4420 degrees of freedom
## Multiple R-squared: 0.1333, Adjusted R-squared: 0.1325
## F-statistic: 169.9 on 4 and 4420 DF, p-value: < 2.2e-16

## BETA: Additive
Addmodel <- Add$model
Addmodel <- sapply(Addmodel, as.numeric)
lm(data.frame(scale(Addmodel)))
## Call:
## lm(formula = data.frame(scale(Addmodel)))
## Coefficients:
## (Intercept) mydf.Additive mydf.Age mydf.SEX
## -3.178e-17 -4.575e-02 3.289e-01 1.569e-01
confint(lm(data.frame(scale(Addmodel))))
## 2.5 % 97.5 %
## (Intercept) -0.02745524 0.02745524
## mydf.Additive -0.07321476 -0.01829363
## mydf.Age 0.30147362 0.35640861
## mydf.SEX 0.12940099 0.18433664

## Result: BMI addiitive model is significant, after adjusting for SEX and Age. r-squared is 0.1325 giving a high heritability.


## BMI and Age DATA FOR TABLE 1:

Additivedf <- data.frame(Additive, mydf[ , "BMI"], mydf[ , "Age"])
colnames(Additivedf) <- c("Additive", "BMI", "Age")
head(Additivedf)

Additivedf_0 <- Additivedf[Additivedf$Additive == 0, ]
head(Additivedf_0)
Additivedf_1 <- Additivedf[Additivedf$Additive == 1, ]
head(Additivedf_1)
Additivedf_2 <- Additivedf[Additivedf$Additive == 2, ]
head(Additivedf_2)

length(Additivedf_0$BMI)
length(Additivedf_1$BMI)
length(Additivedf_2$BMI)
length(Additivedf$BMI)

mean(Additivedf_0$BMI)
mean(Additivedf_1$BMI)
mean(Additivedf_2$BMI)
mean(Additivedf$BMI)

sd(Additivedf_0$BMI)
sd(Additivedf_1$BMI)
sd(Additivedf_2$BMI)
sd(Additivedf$BMI)

median(Additivedf_0$BMI)
median(Additivedf_1$BMI)
median(Additivedf_2$BMI)
median(Additivedf$BMI)

mad(Additivedf_0$BMI, constant = 1)
mad(Additivedf_1$BMI, constant = 1)
mad(Additivedf_2$BMI, constant = 1)
mad(Additivedf$BMI, constant = 1)

length(Additivedf_0$Age)
length(Additivedf_1$Age)
length(Additivedf_2$Age)
length(Additivedf$Age)

mean(Additivedf_0$Age)
mean(Additivedf_1$Age)
mean(Additivedf_2$Age)
mean(Additivedf$Age)

sd(Additivedf_0$Age)
sd(Additivedf_1$Age)
sd(Additivedf_2$Age)
sd(Additivedf$Age)

median(Additivedf_0$Age)
median(Additivedf_1$Age)
median(Additivedf_2$Age)
median(Additivedf$Age)

mad(Additivedf_0$Age, constant = 1)
mad(Additivedf_1$Age, constant = 1)
mad(Additivedf_2$Age, constant = 1)
mad(Additivedf$Age, constant = 1)


## MALES - rs67047829 association with body mass Index.

## MALES ONLY, n = 2933.
MALEdf2 <- mydf2[mydf2$SEX == 1, ]
nrow(MALEdf2)

levels(as.factor(MALEdf2[ , "Age"]))
MALEdf2[ , "Age"] <- as.numeric(MALEdf2[ , "Age"])
sum(is.na(MALEdf2[ , "Age"]))
levels(as.factor(MALEdf2[ , "BMI"]))
MALEdf2[ , "BMI"] <- as.numeric(MALEdf2[ , "BMI"])
sum(is.na(MALEdf2[ , "BMI"]))
levels(as.factor(MALEdf2[ , "SEX"]))
sum(is.na(MALEdf2[ , "SEX"]))

rs67047829 <- paste0(MALEdf2[ , "rs67047829_1"], MALEdf2[ , "rs67047829_2"])
MALEdf2$rs67047829 <- rs67047829
levels(as.factor(MALEdf2[ , "rs67047829"]))
sum(is.na(MALEdf2[ , "rs67047829"]))

## Counts of genotypes - MALEdf2:
levels(as.factor(MALEdf2[ , "rs67047829"]))
GenoMALEdf2 <- MALEdf2[ , "rs67047829"]
GenoMALEdf2_AA <- GenoMALEdf2[GenoMALEdf2 == "AA"]
head(GenoMALEdf2_AA)
length(GenoMALEdf2_AA)
GenoMALEdf2_GG <- GenoMALEdf2[GenoMALEdf2 == "GG"]
head(GenoMALEdf2_GG)
length(GenoMALEdf2_GG)
GenoMALEdf2_GA <- GenoMALEdf2[GenoMALEdf2 == "GA"]
head(GenoMALEdf2_GA)
length(GenoMALEdf2_GA)
GenoMALEdf2_AG <- GenoMALEdf2[GenoMALEdf2 == "AG"]
head(GenoMALEdf2_AG)
length(GenoMALEdf2_AG)

## Remove subject if data missing from Age, BMI, SEX or rs67047829:

MALEdf1 <- MALEdf2[complete.cases(MALEdf2[ , c("Age", "BMI", "SEX", "rs67047829")]), ]
head(MALEdf1)
nrow(MALEdf1)
colnames(MALEdf1)

## Counts of genotypes - MALEdf1
levels(as.factor(MALEdf1[ , "rs67047829"]))
GenoMALEdf1 <- MALEdf1[ , "rs67047829"]
GenoMALEdf1_AA <- GenoMALEdf1[GenoMALEdf1 == "AA"]
head(GenoMALEdf1_AA)
length(GenoMALEdf1_AA)
GenoMALEdf1_GG <- GenoMALEdf1[GenoMALEdf1 == "GG"]
head(GenoMALEdf1_GG)
length(GenoMALEdf1_GG)
GenoMALEdf1_GA <- GenoMALEdf1[GenoMALEdf1 == "GA"]
head(GenoMALEdf1_GA)
length(GenoMALEdf1_GA)
GenoMALEdf1_AG <- GenoMALEdf1[GenoMALEdf1 == "AG"]
head(GenoMALEdf1_AG)
length(GenoMALEdf1_AG)

sum(is.na(MALEdf1[ , "Age"]))
sum(is.na(MALEdf1[ , "BMI"]))
sum(is.na(MALEdf1[ , "SEX"]))
sum(is.na(MALEdf1[ , "rs67047829"]))

## Find subjects with more than a certain percentage of genotype fails:
NoPhendf1 <- MALEdf1[ , 13:ncol(MALEdf1)]
head(NoPhendf1)
ncol(NoPhendf1)
nrow(NoPhendf1)

NAs <- rowSums(is.na(NoPhendf1))
max(NAs)

NAtrue <- list();
for (i in 1:length(NAs)) {
if (NAs[[i]] < 8) { ## <8 missing gives only subjects with <5% missing SNP values
NAtrue[[i]] <- TRUE
} else {
NAtrue[[i]] <- FALSE
}
}
NAtrue <- as.vector(unlist(NAtrue))

MALEdf <- MALEdf1[NAtrue, ]
nrow(MALEdf)
ncol(MALEdf)

MALEdf[ , "BMI"] <- as.numeric(MALEdf[ , "BMI"])
MALEdf[ , "Age"] <- as.numeric(MALEdf[ , "Age"])

## Genotype <- paste0(MALEdf[ , "rs67047829_1"], MALEdf[ , "rs67047829_2"])

levels(as.factor(MALEdf[ , "rs67047829"]))
GenoMALEdf <- MALEdf[ , "rs67047829"]
GenoMALEdf_AA <- GenoMALEdf[GenoMALEdf == "AA"]
head(GenoMALEdf_AA)
length(GenoMALEdf_AA)
GenoMALEdf_GG <- GenoMALEdf[GenoMALEdf == "GG"]
head(GenoMALEdf_GG)
length(GenoMALEdf_GG)
GenoMALEdf_GA <- GenoMALEdf[GenoMALEdf == "GA"]
head(GenoMALEdf_GA)
length(GenoMALEdf_GA)
GenoMALEdf_AG <- GenoMALEdf[GenoMALEdf == "AG"]
head(GenoMALEdf_AG)
length(GenoMALEdf_AG)


Genotype <- MALEdf[ , "rs67047829"] ## Genotype given as e.g. "GG"

levels(as.factor(Genotype))

MALEdf$Genotype <- Genotype
head(MALEdf)

## Dominant model coding:
Dominant <- Genotype
Dominant[(Genotype == "GA") | (Genotype == "AG") | (Genotype == "AA")] <- 1
Dominant[Genotype == "GG"] <- 0
MALEdf$Dominant <- as.factor(Dominant)
## Recessive model coding:
Recessive <- Genotype
Recessive[Genotype == "AA"] <- 0
Recessive[(Genotype == "GA") | (Genotype == "AG") | (Genotype == "GG")] <- 1
MALEdf$Recessive <- as.factor(Recessive)
## Heterozygote model coding:
Heterozygote <- Genotype
Heterozygote[(Genotype == "GA") | (Genotype == "AG")] <- 1
Heterozygote[(Genotype == "GG") | (Genotype == "AA")] <- 0
MALEdf$Heterozygote <- as.factor(Heterozygote)
## Additive model coding:
Additive <- Genotype
Additive[Genotype == "GG"] <- 0
Additive[(Genotype == "GA") | (Genotype == "AG")] <- 1
Additive[Genotype == "AA"] <- 2

MALEdf$Additive <- as.factor(Additive)

levels(as.factor(Genotype))
levels(as.factor(MALEdf[ , "BMI"]))
levels(as.factor(MALEdf[ , "SEX"]))
levels(as.factor(MALEdf[ , "Age"]))
levels(as.factor(Dominant))
levels(as.factor(Recessive))
levels(as.factor(Heterozygote))
levels(as.factor(Additive))

AA <- Genotype[Genotype == "AA"]
AA
AG <- Genotype[Genotype == "AG"]
AG
GA <- Genotype[Genotype == "GA"]
GA
GG <- Genotype[Genotype == "GG"]
GG


## BMI vs. recessive logistic model, adjusted for year of birth.

summary(glm(MALEdf$Recessive ~ MALEdf$BMI + MALEdf$Age, family = binomial, data = MALEdf))

## Call:
## glm(formula = MALEdf$Recessive ~ MALEdf$BMI + MALEdf$Age, family = binomial,
## data = MALEdf)
##
## Deviance Residuals:
## Min 1Q Median 3Q Max
## -3.2374 0.1057 0.1361 0.1626 0.3211
##
## Coefficients:
## Estimate Std. Error z value Pr(>|z|)
## (Intercept) 1.71665 1.30792 1.313 0.1894
## MALEdf$BMI 0.15732 0.06208 2.534 0.0113 *
## MALEdf$Age -0.01909 0.01510 -1.265 0.2060
## ---
## Signif. codes: 0 ‘***’ 0.001 ‘**’ 0.01 ‘*’ 0.05 ‘.’ 0.1 ‘ ’ 1
##
## (Dispersion parameter for binomial family taken to be 1)
##
## Null deviance: 257.35 on 2286 degrees of freedom
## Residual deviance: 249.80 on 2284 degrees of freedom
## AIC: 255.8
##
## Number of Fisher Scoring iterations: 8

## Male result: BMI p value significant, and main effect of age is not significant - and this adjustor is now removed:

## BMI vs. recessive logistic model, adjustment removed.

MALERec <- glm(MALEdf$Recessive ~ MALEdf$BMI, family = binomial, data = MALEdf)
summary(MALERec)

## Call:
## glm(formula = MALEdf$Recessive ~ MALEdf$BMI, family = binomial,
## data = MALEdf)
##
## Deviance Residuals:
## Min 1Q Median 3Q Max
## -3.2852 0.1096 0.1391 0.1634 0.2583
##
## Coefficients:
## Estimate Std. Error z value Pr(>|z|)
## (Intercept) 1.59456 1.28985 1.236 0.2164
## MALEdf$BMI 0.12768 0.05671 2.252 0.0243 *
## ---
## Signif. codes: 0 ‘***’ 0.001 ‘**’ 0.01 ‘*’ 0.05 ‘.’ 0.1 ‘ ’ 1
##
## (Dispersion parameter for binomial family taken to be 1)
##
## Null deviance: 257.35 on 2286 degrees of freedom
## Residual deviance: 251.35 on 2285 degrees of freedom
## AIC: 255.35
##
## Number of Fisher Scoring iterations: 8

## BETA: MALE Recessive
MALERecmodel <- MALERec$model
MALERecmodel <- as.data.frame(sapply(MALERecmodel, as.numeric))
MALERecScale1 <- data.frame(scale(MALERecmodel[ , 2]))
MALERecScale <- cbind(MALERecmodel[ , 1], MALERecScale1)
colnames(MALERecScale) <- c("MALE_Recessive", "BMIScale")
MALERecScale$MALE_Recessive <- as.factor(MALERecScale$MALE_Recessive)
levels(MALERecScale$MALE_Recessive) <- list("0" = "1", "1" = "2")

MALERecScale.glm <- glm(MALERecScale$MALE_Recessive ~ MALERecScale$BMI, family = binomial, data = MALERecScale)
summary(MALERecScale.glm)

## Call:
## glm(formula = MALERecScale$MALE_Recessive ~ MALERecScale$BMI,
## family = binomial, data = MALERecScale)
##
## Deviance Residuals:
## Min 1Q Median 3Q Max
## -3.2852 0.1096 0.1391 0.1634 0.2583
##
## Coefficients:
## Estimate Std. Error z value Pr(>|z|)
## (Intercept) 4.7439 0.2448 19.379 <2e-16 ***
## MALERecScale$BMI 0.6181 0.2745 2.252 0.0243 *
## ---
## Signif. codes: 0 ‘***’ 0.001 ‘**’ 0.01 ‘*’ 0.05 ‘.’ 0.1 ‘ ’ 1
##
## (Dispersion parameter for binomial family taken to be 1)
##
## Null deviance: 257.35 on 2286 degrees of freedom
## Residual deviance: 251.35 on 2285 degrees of freedom
## AIC: 255.35
##
## Number of Fisher Scoring iterations: 8

confint(MALERecScale.glm)
## 2.5 % 97.5 %
## (Intercept) 4.3059347 5.276075
## MALERecScale$BMI 0.1146408 1.190371

## Male result: Final BMI p value for the recessive model - significant.


## BMI vs. dominant model, adjusted for year of birth.

summary(glm(MALEdf$Dominant ~ MALEdf$BMI + MALEdf$Age, family = binomial, data = MALEdf))

## Call:
## glm(formula = MALEdf$Dominant ~ MALEdf$BMI + MALEdf$Age, family = binomial,
## data = MALEdf)
##
## Deviance Residuals:
## Min 1Q Median 3Q Max
## -0.7394 -0.6507 -0.6203 -0.5623 2.1022
##
## Coefficients:
## Estimate Std. Error z value Pr(>|z|)
## (Intercept) -0.776201 0.298567 -2.600 0.00933 **
## MALEdf$BMI -0.034424 0.013171 -2.614 0.00896 **
## MALEdf$Age 0.002189 0.004040 0.542 0.58806
## ---
## Signif. codes: 0 ‘***’ 0.001 ‘**’ 0.01 ‘*’ 0.05 ‘.’ 0.1 ‘ ’ 1
##
## (Dispersion parameter for binomial family taken to be 1)
##
## Null deviance: 2148.1 on 2286 degrees of freedom
## Residual deviance: 2140.6 on 2284 degrees of freedom
## AIC: 2146.6
##
## Number of Fisher Scoring iterations: 4
##
## Male result. BMI p-value for Dominant model much lower than recessive model with adjustor. ## Main effect of Age not significant and this adjustor is removed below:


## BMI vs. dominant model, adjustors removed.

MALEDom <- glm(MALEdf$Dominant ~ MALEdf$BMI, family = binomial, data = MALEdf)
summary(MALEDom)

## Call:
## glm(formula = MALEdf$Dominant ~ MALEdf$BMI, family = binomial,
## data = MALEdf)
##
## Deviance Residuals:
## Min 1Q Median 3Q Max
## -0.7270 -0.6509 -0.6202 -0.5629 2.1017
##
## Coefficients:
## Estimate Std. Error z value Pr(>|z|)
## (Intercept) -0.75437 0.29512 -2.556 0.01058 *
## MALEdf$BMI -0.03151 0.01198 -2.630 0.00854 **
## ---
## Signif. codes: 0 ‘***’ 0.001 ‘**’ 0.01 ‘*’ 0.05 ‘.’ 0.1 ‘ ’ 1
##
## (Dispersion parameter for binomial family taken to be 1)
##
## Null deviance: 2148.1 on 2286 degrees of freedom
## Residual deviance: 2140.9 on 2285 degrees of freedom
## AIC: 2144.9
##
## Number of Fisher Scoring iterations: 4

## BETA: MALE Dominant
MALEDommodel <- MALEDom$model
MALEDommodel <- as.data.frame(sapply(MALEDommodel, as.numeric))
MALEDomScale1 <- data.frame(scale(MALEDommodel[ , 2]))
MALEDomScale <- cbind(MALEDommodel[ , 1], MALEDomScale1)
colnames(MALEDomScale) <- c("MALE_Dominant", "BMIScale")
MALEDomScale$MALE_Dominant <- as.factor(MALEDomScale$MALE_Dominant)
levels(MALEDomScale$MALE_Dominant) <- list("0" = "1", "1" = "2")

MALEDomScale.glm <- glm(MALEDomScale$MALE_Dominant ~ MALEDomScale$BMI, family = binomial, data = MALEDomScale)
summary(MALEDomScale.glm)

## Call:
## glm(formula = MALEDomScale$MALE_Dominant ~ MALEDomScale$BMI,
## family = binomial, data = MALEDomScale)
##
## Deviance Residuals:
## Min 1Q Median 3Q Max
## -0.7270 -0.6509 -0.6202 -0.5629 2.1017
##
## Coefficients:
## Estimate Std. Error z value Pr(>|z|)
## (Intercept) -1.53159 0.05493 -27.88 < 2e-16 ***
## MALEDomScale$BMI -0.15253 0.05800 -2.63 0.00854 **
## ---
## Signif. codes: 0 ‘***’ 0.001 ‘**’ 0.01 ‘*’ 0.05 ‘.’ 0.1 ‘ ’ 1
##
## (Dispersion parameter for binomial family taken to be 1)
##
## Null deviance: 2148.1 on 2286 degrees of freedom
## Residual deviance: 2140.9 on 2285 degrees of freedom
## AIC: 2144.9
##
## Number of Fisher Scoring iterations: 4

confint(MALEDomScale.glm)
## 2.5 % 97.5 %
## (Intercept) -1.6405486 -1.42517281
## MALEDomScale$BMI -0.2680055 -0.04058522


## Male result: Final low BMI p value for dominant model is lower than for recessive model.


## BMI vs. heterozygote model, adjusted for year of birth.

summary(glm(MALEdf$Heterozygote ~ MALEdf$BMI + MALEdf$Age, family = binomial, data = MALEdf))

## Call:
## glm(formula = MALEdf$Heterozygote ~ MALEdf$BMI + MALEdf$Age,
## family = binomial, data = MALEdf)
##
## Deviance Residuals:
## Min 1Q Median 3Q Max
## -0.6887 -0.6270 -0.6034 -0.5598 2.0846
##
## Coefficients:
## Estimate Std. Error z value Pr(>|z|)
## (Intercept) -0.982118 0.303002 -3.241 0.00119 **
## MALEdf$BMI -0.026753 0.013306 -2.011 0.04436 *
## MALEdf$Age 0.001001 0.004133 0.242 0.80864
## ---
## Signif. codes: 0 ‘***’ 0.001 ‘**’ 0.01 ‘*’ 0.05 ‘.’ 0.1 ‘ ’ 1
##
## (Dispersion parameter for binomial family taken to be 1)
##
## Null deviance: 2076.4 on 2286 degrees of freedom
## Residual deviance: 2071.7 on 2284 degrees of freedom
## AIC: 2077.7
##
## Number of Fisher Scoring iterations: 4
##
## Male result. Heterozygote model significant with adjustment for Age - adjustor main effect not significant - and can be removed:


## BMI vs. heterozygote model, adjustor removed.

MALEHet <- glm(MALEdf$Heterozygote ~ MALEdf$BMI, family = binomial, data = MALEdf)
summary(MALEHet)

## Call:
## glm(formula = MALEdf$Heterozygote ~ MALEdf$BMI, family = binomial,
## data = MALEdf)
##
## Deviance Residuals:
## Min 1Q Median 3Q Max
## -0.6856 -0.6268 -0.6036 -0.5594 2.0846
##
## Coefficients:
## Estimate Std. Error z value Pr(>|z|)
## (Intercept) -0.97182 0.29966 -3.243 0.00118 **
## MALEdf$BMI -0.02544 0.01212 -2.098 0.03591 *
## ---
## Signif. codes: 0 ‘***’ 0.001 ‘**’ 0.01 ‘*’ 0.05 ‘.’ 0.1 ‘ ’ 1
##
## (Dispersion parameter for binomial family taken to be 1)
##
## Null deviance: 2076.4 on 2286 degrees of freedom
## Residual deviance: 2071.8 on 2285 degrees of freedom
## AIC: 2075.8
##
## Number of Fisher Scoring iterations: 4


## BETA: MALE Heterozygote
MALEHetmodel <- MALEHet$model
MALEHetmodel <- as.data.frame(sapply(MALEHetmodel, as.numeric))
MALEHetScale1 <- data.frame(scale(MALEHetmodel[ , 2]))
MALEHetScale <- cbind(MALEHetmodel[ , 1], MALEHetScale1)
colnames(MALEHetScale) <- c("MALE_Heterozygote", "BMIScale")
MALEHetScale$MALE_Heterozygote <- as.factor(MALEHetScale$MALE_Heterozygote)
levels(MALEHetScale$MALE_Heterozygote) <- list("0" = "1", "1" = "2")

MALEHetScale.glm <- glm(MALEHetScale$MALE_Heterozygote ~ MALEHetScale$BMI, family = binomial, data = MALEHetScale)
summary(MALEHetScale.glm)

## Call:
## glm(formula = MALEHetScale$MALE_Heterozygote ~ MALEHetScale$BMI,
## family = binomial, data = MALEHetScale)
##
## Deviance Residuals:
## Min 1Q Median 3Q Max
## -0.6856 -0.6268 -0.6036 -0.5594 2.0846
##
## Coefficients:
## Estimate Std. Error z value Pr(>|z|)
## (Intercept) -1.59924 0.05608 -28.519 <2e-16 ***
## MALEHetScale$BMI -0.12313 0.05869 -2.098 0.0359 *
## ---
## Signif. codes: 0 ‘***’ 0.001 ‘**’ 0.01 ‘*’ 0.05 ‘.’ 0.1 ‘ ’ 1
##
## (Dispersion parameter for binomial family taken to be 1)
##
## Null deviance: 2076.4 on 2286 degrees of freedom
## Residual deviance: 2071.8 on 2285 degrees of freedom
## AIC: 2075.8
##
## Number of Fisher Scoring iterations: 4

confint(MALEHetScale.glm)
## 2.5 % 97.5 %
## (Intercept) -1.7105391 -1.490657565
## MALEHetScale$BMI -0.2400294 -0.009885912


## Male result. Heterozygote model is significant.

## BMI vs. additive model, adjusted for year of birth - linear regression.

Additive <- as.factor(Additive)

## I The coefficient of determination (r 2 ) of an additive linear regression model gives an estimate of the proportion of phenotypic variation that is explained by the SNP (or SNPs) in the model, e.g., the ”SNP heritability”
## http://faculty.washington.edu/tathornt/SISG2017/lectures/SISG2017session02.pdf

## Anova(lm(MALEdf$BMI ~ MALEdf$Additive + MALEdf$Age), type = "II", white.adjust = TRUE)
##
## Coefficient covariances computed by hccm()
## Analysis of Deviance Table (Type II tests)
##
## Response: MALEdf$BMI
## Df F Pr(>F)
## MALEdf$Additive 2 7.8007 0.0004204 ***
## MALEdf$Age 1 447.0157 < 2.2e-16 ***
## Residuals 2283
## ---
## Signif. codes: 0 ‘***’ 0.001 ‘**’ 0.01 ‘*’ 0.05 ‘.’ 0.1 ‘ ’ 1
##
## Male result: additive model is highly significant - but Age main effects is also very significant - therefore assess interaction:

Anova(lm(MALEdf$BMI ~ MALEdf$Additive * MALEdf$Age), type = "II", white.adjust = TRUE)

## Coefficient covariances computed by hccm()
## Analysis of Deviance Table (Type II tests)
##
## Response: MALEdf$BMI
## Df F Pr(>F)
## MALEdf$Additive 2 8.1399 0.0003002 ***
## MALEdf$Age 1 444.4754 < 2.2e-16 ***
## MALEdf$Additive:MALEdf$Age 2 1.5387 0.2148906
## Residuals 2281
## ---
## Signif. codes: 0 ‘***’ 0.001 ‘**’ 0.01 ‘*’ 0.05 ‘.’ 0.1 ‘ ’ 1
##

## Male result: Age interaction with BMI is not significant - therefore remove interaction, but not adjustor:

Anova(lm(MALEdf$BMI ~ MALEdf$Additive + MALEdf$Age), type = "II", white.adjust = TRUE)

## Coefficient covariances computed by hccm()
## Analysis of Deviance Table (Type II tests)
##
## Response: MALEdf$BMI
## Df F Pr(>F)
## MALEdf$Additive 2 7.8007 0.0004204 ***
## MALEdf$Age 1 447.0157 < 2.2e-16 ***
## Residuals 2283
## ---
## Signif. codes: 0 ‘***’ 0.001 ‘**’ 0.01 ‘*’ 0.05 ‘.’ 0.1 ‘ ’ 1
##
##
## Male result: final BMI p value for additive model.

MALEAdd <- lm(MALEdf$BMI ~ MALEdf$Additive + MALEdf$Age)
summary(MALEAdd)

## Call:
## lm(formula = MALEdf$BMI ~ MALEdf$Additive + MALEdf$Age)
##
## Residuals:
## Min 1Q Median 3Q Max
## -11.986 -3.021 -0.755 2.405 36.076
##
## Coefficients:
## Estimate Std. Error t value Pr(>|t|)
## (Intercept) 19.132737 0.284458 67.260 < 2e-16 ***
## MALEdf$Additive1 -0.524337 0.246741 -2.125 0.03369 *
## MALEdf$Additive2 -2.397842 0.926156 -2.589 0.00969 **
## MALEdf$Age 0.132333 0.006212 21.304 < 2e-16 ***
## ---
## Signif. codes: 0 ‘***’ 0.001 ‘**’ 0.01 ‘*’ 0.05 ‘.’ 0.1 ‘ ’ 1
##
## Residual standard error: 4.415 on 2283 degrees of freedom
## Multiple R-squared: 0.1694, Adjusted R-squared: 0.1683
## F-statistic: 155.2 on 3 and 2283 DF, p-value: < 2.2e-16

## BETA: MALE Additive
MALEAddmodel <- MALEAdd$model
MALEAddmodel <- sapply(MALEAddmodel, as.numeric)
lm(data.frame(scale(MALEAddmodel)))
## Call:
## lm(formula = data.frame(scale(MALEAddmodel)))
## Coefficients:
## (Intercept) MALEdf.Additive MALEdf.Age
## -6.159e-17 -5.730e-02 4.062e-01
confint(lm(data.frame(scale(MALEAddmodel))))
## 2.5 % 97.5 %
## (Intercept) -0.03740152 0.03740152
## MALEdf.Additive -0.09471345 -0.01988930
## MALEdf.Age 0.36877436 0.44359851

## Male result: BMI addiitive model is significant, after adjusting for Age. r-squared is 0.1683 giving a high heritability.

## BMI and Age DATA FOR TABLE 1: MALES

Additivedf <- data.frame(Additive, MALEdf[ , "BMI"], MALEdf[ , "Age"])
colnames(Additivedf) <- c("Additive", "BMI", "Age")
head(Additivedf)

Additivedf_0 <- Additivedf[Additivedf$Additive == 0, ]
head(Additivedf_0)
Additivedf_1 <- Additivedf[Additivedf$Additive == 1, ]
head(Additivedf_1)
Additivedf_2 <- Additivedf[Additivedf$Additive == 2, ]
head(Additivedf_2)

length(Additivedf_0$BMI)
length(Additivedf_1$BMI)
length(Additivedf_2$BMI)
length(Additivedf$BMI)

mean(Additivedf_0$BMI)
mean(Additivedf_1$BMI)
mean(Additivedf_2$BMI)
mean(Additivedf$BMI)

sd(Additivedf_0$BMI)
sd(Additivedf_1$BMI)
sd(Additivedf_2$BMI)
sd(Additivedf$BMI)

median(Additivedf_0$BMI)
median(Additivedf_1$BMI)
median(Additivedf_2$BMI)
median(Additivedf$BMI)

mad(Additivedf_0$BMI, constant = 1)
mad(Additivedf_1$BMI, constant = 1)
mad(Additivedf_2$BMI, constant = 1)
mad(Additivedf$BMI, constant = 1)

length(Additivedf_0$Age)
length(Additivedf_1$Age)
length(Additivedf_2$Age)
length(Additivedf$Age)

mean(Additivedf_0$Age)
mean(Additivedf_1$Age)
mean(Additivedf_2$Age)
mean(Additivedf$Age)

sd(Additivedf_0$Age)
sd(Additivedf_1$Age)
sd(Additivedf_2$Age)
sd(Additivedf$Age)

median(Additivedf_0$Age)
median(Additivedf_1$Age)
median(Additivedf_2$Age)
median(Additivedf$Age)

mad(Additivedf_0$Age, constant = 1)
mad(Additivedf_1$Age, constant = 1)
mad(Additivedf_2$Age, constant = 1)
mad(Additivedf$Age, constant = 1)


## FEMALES - rs67047829 association with body mass Index.

## FEMALES ONLY, N = 2824.
femdf2 <- mydf2[mydf2$SEX == 2, ]
nrow(femdf2)

levels(as.factor(femdf2[ , "Age"]))
femdf2[ , "Age"] <- as.numeric(femdf2[ , "Age"])
sum(is.na(femdf2[ , "Age"]))
levels(as.factor(femdf2[ , "BMI"]))
femdf2[ , "BMI"] <- as.numeric(femdf2[ , "BMI"])
sum(is.na(femdf2[ , "BMI"]))
levels(as.factor(femdf2[ , "SEX"]))
sum(is.na(femdf2[ , "SEX"]))

rs67047829 <- paste0(femdf2[ , "rs67047829_1"], femdf2[ , "rs67047829_2"])
femdf2$rs67047829 <- rs67047829
levels(as.factor(femdf2[ , "rs67047829"]))
sum(is.na(femdf2[ , "rs67047829"]))

## Counts of genotypes - femdf2:
levels(as.factor(femdf2[ , "rs67047829"]))
Genofemdf2 <- femdf2[ , "rs67047829"]
Genofemdf2_AA <- Genofemdf2[Genofemdf2 == "AA"]
head(Genofemdf2_AA)
length(Genofemdf2_AA)
Genofemdf2_GG <- Genofemdf2[Genofemdf2 == "GG"]
head(Genofemdf2_GG)
length(Genofemdf2_GG)
Genofemdf2_GA <- Genofemdf2[Genofemdf2 == "GA"]
head(Genofemdf2_GA)
length(Genofemdf2_GA)
Genofemdf2_AG <- Genofemdf2[Genofemdf2 == "AG"]
head(Genofemdf2_AG)
length(Genofemdf2_AG)

## Remove subject if data missing from Age, BMI, SEX or rs67047829:

femdf1 <- femdf2[complete.cases(femdf2[ , c("Age", "BMI", "SEX", "rs67047829")]), ]
head(femdf1)
nrow(femdf1)
colnames(femdf1)

## Counts of genotypes - femdf1
levels(as.factor(femdf1[ , "rs67047829"]))
Genofemdf1 <- femdf1[ , "rs67047829"]
Genofemdf1_AA <- Genofemdf1[Genofemdf1 == "AA"]
head(Genofemdf1_AA)
length(Genofemdf1_AA)
Genofemdf1_GG <- Genofemdf1[Genofemdf1 == "GG"]
head(Genofemdf1_GG)
length(Genofemdf1_GG)
Genofemdf1_GA <- Genofemdf1[Genofemdf1 == "GA"]
head(Genofemdf1_GA)
length(Genofemdf1_GA)
Genofemdf1_AG <- Genofemdf1[Genofemdf1 == "AG"]
head(Genofemdf1_AG)
length(Genofemdf1_AG)

sum(is.na(femdf1[ , "Age"]))
sum(is.na(femdf1[ , "BMI"]))
sum(is.na(femdf1[ , "SEX"]))
sum(is.na(femdf1[ , "rs67047829"]))

## Find subjects with more than a certain percentage of genotype fails:
NoPhendf1 <- femdf1[ , 13:ncol(femdf1)]
head(NoPhendf1)
ncol(NoPhendf1)
nrow(NoPhendf1)

NAs <- rowSums(is.na(NoPhendf1))
max(NAs)

NAtrue <- list();
for (i in 1:length(NAs)) {
if (NAs[[i]] < 8) { ## <8 missing gives <5% missing SNP values
NAtrue[[i]] <- TRUE
} else {
NAtrue[[i]] <- FALSE
}
}
NAtrue <- as.vector(unlist(NAtrue))

femdf <- femdf1[NAtrue, ]
nrow(femdf)
ncol(femdf)

femdf[ , "BMI"] <- as.numeric(femdf[ , "BMI"])
femdf[ , "Age"] <- as.numeric(femdf[ , "Age"])

## Genotype <- paste0(femdf[ , "rs67047829_1"], femdf[ , "rs67047829_2"])

levels(as.factor(femdf[ , "rs67047829"]))
Genofemdf <- femdf[ , "rs67047829"]
Genofemdf_AA <- Genofemdf[Genofemdf == "AA"]
head(Genofemdf_AA)
length(Genofemdf_AA)
Genofemdf_GG <- Genofemdf[Genofemdf == "GG"]
head(Genofemdf_GG)
length(Genofemdf_GG)
Genofemdf_GA <- Genofemdf[Genofemdf == "GA"]
head(Genofemdf_GA)
length(Genofemdf_GA)
Genofemdf_AG <- Genofemdf[Genofemdf == "AG"]
head(Genofemdf_AG)
length(Genofemdf_AG)


Genotype <- femdf[ , "rs67047829"] ## Genotype given as e.g. "GG"

levels(as.factor(Genotype))

femdf$Genotype <- Genotype
head(femdf)
## Dominant model coding:
Dominant <- Genotype
Dominant[(Genotype == "GA") | (Genotype == "AG") | (Genotype == "AA")] <- 1
Dominant[Genotype == "GG"] <- 0
femdf$Dominant <- as.factor(Dominant)
## Recessive model coding:
Recessive <- Genotype
Recessive[Genotype == "AA"] <- 0
Recessive[(Genotype == "GA") | (Genotype == "AG") | (Genotype == "GG")] <- 1
femdf$Recessive <- as.factor(Recessive)
## Heterozygote model coding:
Heterozygote <- Genotype
Heterozygote[(Genotype == "GA") | (Genotype == "AG")] <- 1
Heterozygote[(Genotype == "GG") | (Genotype == "AA")] <- 0
femdf$Heterozygote <- as.factor(Heterozygote)
## Additive model coding:
Additive <- Genotype
Additive[Genotype == "GG"] <- 0
Additive[(Genotype == "GA") | (Genotype == "AG")] <- 1
Additive[Genotype == "AA"] <- 2

femdf$Additive <- as.factor(Additive)

levels(as.factor(Genotype))
levels(as.factor(femdf[ , "BMI"]))
levels(as.factor(femdf[ , "SEX"]))
levels(as.factor(femdf[ , "Age"]))
levels(as.factor(Dominant))
levels(as.factor(Recessive))
levels(as.factor(Heterozygote))
levels(as.factor(Additive))

AA <- Genotype[Genotype == "AA"]
AA
AG <- Genotype[Genotype == "AG"]
AG
GA <- Genotype[Genotype == "GA"]
GA
GG <- Genotype[Genotype == "GG"]
GG


## BMI vs. recessive logistic model, adjusted for year of birth.

summary(glm(femdf$Recessive ~ femdf$BMI + femdf$Age, family = binomial, data = femdf))

## Call:
## glm(formula = femdf$Recessive ~ femdf$BMI + femdf$Age, family = binomial,
## data = femdf)
##
## Deviance Residuals:
## Min 1Q Median 3Q Max
## -3.2250 0.1174 0.1371 0.1565 0.2401
##
## Coefficients:
## Estimate Std. Error z value Pr(>|z|)
## (Intercept) 1.845898 1.531034 1.206 0.2280
## femdf$BMI 0.106530 0.063463 1.679 0.0932 .
## femdf$Age 0.001967 0.015525 0.127 0.8992
## ---
## Signif. codes: 0 ‘***’ 0.001 ‘**’ 0.01 ‘*’ 0.05 ‘.’ 0.1 ‘ ’ 1
##
## (Dispersion parameter for binomial family taken to be 1)
##
## Null deviance: 235.96 on 2137 degrees of freedom
## Residual deviance: 232.56 on 2135 degrees of freedom
## AIC: 238.56
##
## Number of Fisher Scoring iterations: 7


## Female result: BMI p value not significant, and main effect of age is not significant - and this adjustor is now removed:

## BMI vs. recessive logistic model, adjustment removed.

femRec <- glm(femdf$Recessive ~ femdf$BMI, family = binomial, data = femdf)
summary(femRec)

## Call:
## glm(formula = femdf$Recessive ~ femdf$BMI, family = binomial,
## data = femdf)
##
## Deviance Residuals:
## Min 1Q Median 3Q Max
## -3.2199 0.1171 0.1372 0.1564 0.2376
##
## Coefficients:
## Estimate Std. Error z value Pr(>|z|)
## (Intercept) 1.87361 1.51780 1.234 0.2170
## femdf$BMI 0.10863 0.06134 1.771 0.0766 .
## ---
## Signif. codes: 0 ‘***’ 0.001 ‘**’ 0.01 ‘*’ 0.05 ‘.’ 0.1 ‘ ’ 1
##
## (Dispersion parameter for binomial family taken to be 1)
##
## Null deviance: 235.96 on 2137 degrees of freedom
## Residual deviance: 232.57 on 2136 degrees of freedom
## AIC: 236.57
##
## Number of Fisher Scoring iterations: 7

## BETA: female Recessive
femRecmodel <- femRec$model
femRecmodel <- as.data.frame(sapply(femRecmodel, as.numeric))
femRecScale1 <- data.frame(scale(femRecmodel[ , 2]))
femRecScale <- cbind(femRecmodel[ , 1], femRecScale1)
colnames(femRecScale) <- c("fem_Recessive", "BMIScale")
femRecScale$fem_Recessive <- as.factor(femRecScale$fem_Recessive)
levels(femRecScale$fem_Recessive) <- list("0" = "1", "1" = "2")

femRecScale.glm <- glm(femRecScale$fem_Recessive ~ femRecScale$BMI, family = binomial, data = femRecScale)
summary(femRecScale.glm)

## Call:
## glm(formula = femRecScale$fem_Recessive ~ femRecScale$BMI, family = binomial,
## data = femRecScale)
##
## Deviance Residuals:
## Min 1Q Median 3Q Max
## -3.2199 0.1171 0.1372 0.1564 0.2376
##
## Coefficients:
## Estimate Std. Error z value Pr(>|z|)
## (Intercept) 4.7004 0.2388 19.679 <2e-16 ***
## femRecScale$BMI 0.4420 0.2496 1.771 0.0766 .
## ---
## Signif. codes: 0 ‘***’ 0.001 ‘**’ 0.01 ‘*’ 0.05 ‘.’ 0.1 ‘ ’ 1
##
## (Dispersion parameter for binomial family taken to be 1)
##
## Null deviance: 235.96 on 2137 degrees of freedom
## Residual deviance: 232.57 on 2136 degrees of freedom
## AIC: 236.57
##
## Number of Fisher Scoring iterations: 7

confint(femRecScale.glm)
## 2.5 % 97.5 %
## (Intercept) 4.26918561 5.2142235
## femRecScale$BMI -0.02719855 0.9495762

## Female result: Final BMI p value for the recessive model - not significant.

## BMI vs. dominant model, adjusted for year of birth.

summary(glm(femdf$Dominant ~ femdf$BMI + femdf$Age, family = binomial, data = femdf))

## Call:
## glm(formula = femdf$Dominant ~ femdf$BMI + femdf$Age, family = binomial,
## data = femdf)
##
## Deviance Residuals:
## Min 1Q Median 3Q Max
## -0.6648 -0.6233 -0.6092 -0.5869 1.9971
##
## Coefficients:
## Estimate Std. Error z value Pr(>|z|)
## (Intercept) -1.1471118 0.3837484 -2.989 0.0028 **
## femdf$BMI -0.0178138 0.0148319 -1.201 0.2297
## femdf$Age 0.0007577 0.0040371 0.188 0.8511
## ---
## Signif. codes: 0 ‘***’ 0.001 ‘**’ 0.01 ‘*’ 0.05 ‘.’ 0.1 ‘ ’ 1
##
## (Dispersion parameter for binomial family taken to be 1)
##
## Null deviance: 1957.4 on 2137 degrees of freedom
## Residual deviance: 1955.9 on 2135 degrees of freedom
## AIC: 1961.9
##
## Number of Fisher Scoring iterations: 4

## Female result. BMI p-value for Dominant model not significant with adjustor. Main effect of Age not significant and this adjustor is removed below:


## BMI vs. dominant model, adjustors removed.

femDom <- glm(femdf$Dominant ~ femdf$BMI, family = binomial, data = femdf)
summary(femDom)

## Call:
## glm(formula = femdf$Dominant ~ femdf$BMI, family = binomial,
## data = femdf)
##
## Deviance Residuals:
## Min 1Q Median 3Q Max
## -0.6646 -0.6228 -0.6085 -0.5871 2.0012
##
## Coefficients:
## Estimate Std. Error z value Pr(>|z|)
## (Intercept) -1.13287 0.37590 -3.014 0.00258 **
## femdf$BMI -0.01714 0.01438 -1.192 0.23330
## ---
## Signif. codes: 0 ‘***’ 0.001 ‘**’ 0.01 ‘*’ 0.05 ‘.’ 0.1 ‘ ’ 1
##
## (Dispersion parameter for binomial family taken to be 1)
##
## Null deviance: 1957.4 on 2137 degrees of freedom
## Residual deviance: 1956.0 on 2136 degrees of freedom
## AIC: 1960
##
## Number of Fisher Scoring iterations: 4


## BETA: female Dominant
femDommodel <- femDom$model
femDommodel <- as.data.frame(sapply(femDommodel, as.numeric))
femDomScale1 <- data.frame(scale(femDommodel[ , 2]))
femDomScale <- cbind(femDommodel[ , 1], femDomScale1)
colnames(femDomScale) <- c("fem_Dominant", "BMIScale")
femDomScale$fem_Dominant <- as.factor(femDomScale$fem_Dominant)
levels(femDomScale$fem_Dominant) <- list("0" = "1", "1" = "2")

femDomScale.glm <- glm(femDomScale$fem_Dominant ~ femDomScale$BMI, family = binomial, data = femDomScale)
summary(femDomScale.glm)

## Call:
## glm(formula = femDomScale$fem_Dominant ~ femDomScale$BMI, family = binomial,
## data = femDomScale)
##
## Deviance Residuals:
## Min 1Q Median 3Q Max
## -0.6646 -0.6228 -0.6085 -0.5871 2.0012
##
## Coefficients:
## Estimate Std. Error z value Pr(>|z|)
## (Intercept) -1.57882 0.05750 -27.459 <2e-16 ***
## femDomScale$BMI -0.06973 0.05851 -1.192 0.233
## ---
## Signif. codes: 0 ‘***’ 0.001 ‘**’ 0.01 ‘*’ 0.05 ‘.’ 0.1 ‘ ’ 1
##
## (Dispersion parameter for binomial family taken to be 1)
##
## Null deviance: 1957.4 on 2137 degrees of freedom
## Residual deviance: 1956.0 on 2136 degrees of freedom
## AIC: 1960
##
## Number of Fisher Scoring iterations: 4

confint(femDomScale.glm)
## 2.5 % 97.5 %
## (Intercept) -1.6929479 -1.46749652
## femDomScale$BMI -0.1856515 0.04377057


## Female result: Final p value for dominant model not significant.


## BMI vs. heterozygote model, adjusted for year of birth.

summary(glm(femdf$Heterozygote ~ femdf$BMI + femdf$Age, family = binomial, data = femdf))

## Call:
## glm(formula = femdf$Heterozygote ~ femdf$BMI + femdf$Age, family = binomial,
## data = femdf)
##
## Deviance Residuals:
## Min 1Q Median 3Q Max
## -0.6285 -0.6001 -0.5914 -0.5774 1.9826
##
## Coefficients:
## Estimate Std. Error z value Pr(>|z|)
## (Intercept) -1.3822278 0.3917117 -3.529 0.000418 ***
## femdf$BMI -0.0118302 0.0150849 -0.784 0.432899
## femdf$Age 0.0009827 0.0041312 0.238 0.811987
## ---
## Signif. codes: 0 ‘***’ 0.001 ‘**’ 0.01 ‘*’ 0.05 ‘.’ 0.1 ‘ ’ 1
##
## (Dispersion parameter for binomial family taken to be 1)
##
## Null deviance: 1889.7 on 2137 degrees of freedom
## Residual deviance: 1889.1 on 2135 degrees of freedom
## AIC: 1895.1
##
## Number of Fisher Scoring iterations: 3

## Female result. Heterozygote model not significant with adjustment for Age - adjustor main effect not significant - and can be removed:


## BMI vs. heterozygote model, adjustor removed.

femHet <- glm(femdf$Heterozygote ~ femdf$BMI, family = binomial, data = femdf)
summary(femHet)

## Call:
## glm(formula = femdf$Heterozygote ~ femdf$BMI, family = binomial,
## data = femdf)
##
## Deviance Residuals:
## Min 1Q Median 3Q Max
## -0.6253 -0.5998 -0.5913 -0.5780 1.9880
##
## Coefficients:
## Estimate Std. Error z value Pr(>|z|)
## (Intercept) -1.36349 0.38333 -3.557 0.000375 ***
## femdf$BMI -0.01096 0.01463 -0.750 0.453484
## ---
## Signif. codes: 0 ‘***’ 0.001 ‘**’ 0.01 ‘*’ 0.05 ‘.’ 0.1 ‘ ’ 1
##
## (Dispersion parameter for binomial family taken to be 1)
##
## Null deviance: 1889.7 on 2137 degrees of freedom
## Residual deviance: 1889.1 on 2136 degrees of freedom
## AIC: 1893.1
##
## Number of Fisher Scoring iterations: 3

## BETA: female Heterozygote
femHetmodel <- femHet$model
femHetmodel <- as.data.frame(sapply(femHetmodel, as.numeric))
femHetScale1 <- data.frame(scale(femHetmodel[ , 2]))
femHetScale <- cbind(femHetmodel[ , 1], femHetScale1)
colnames(femHetScale) <- c("fem_Heterozygote", "BMIScale")
femHetScale$fem_Heterozygote <- as.factor(femHetScale$fem_Heterozygote)
levels(femHetScale$fem_Heterozygote) <- list("0" = "1", "1" = "2")

femHetScale.glm <- glm(femHetScale$fem_Heterozygote ~ femHetScale$BMI, family = binomial, data = femHetScale)
summary(femHetScale.glm)

## Call:
## glm(formula = femHetScale$fem_Heterozygote ~ femHetScale$BMI,
## family = binomial, data = femHetScale)
##
## Deviance Residuals:
## Min 1Q Median 3Q Max
## -0.6253 -0.5998 -0.5913 -0.5780 1.9880
##
## Coefficients:
## Estimate Std. Error z value Pr(>|z|)
## (Intercept) -1.64877 0.05882 -28.03 <2e-16 ***
## femHetScale$BMI -0.04461 0.05951 -0.75 0.453
## ---
## Signif. codes: 0 ‘***’ 0.001 ‘**’ 0.01 ‘*’ 0.05 ‘.’ 0.1 ‘ ’ 1
##
## (Dispersion parameter for binomial family taken to be 1)
##
## Null deviance: 1889.7 on 2137 degrees of freedom
## Residual deviance: 1889.1 on 2136 degrees of freedom
## AIC: 1893.1
##
## Number of Fisher Scoring iterations: 3

confint(femHetScale.glm)
## 2.5 % 97.5 %
## (Intercept) -1.7656105 -1.5349466
## femHetScale$BMI -0.1625456 0.0708369


## Female result. Heterozygote model is not significant.

## BMI vs. additive model, adjusted for year of birth - linear regression.

Additive <- as.factor(Additive)

## I The coefficient of determination (r 2 ) of an additive linear regression model gives an estimate of the proportion of phenotypic variation that is explained by the SNP (or SNPs) in the model, e.g., the ”SNP heritability”
## http://faculty.washington.edu/tathornt/SISG2017/lectures/SISG2017session02.pdf

femAdd <- Anova(lm(femdf$BMI ~ femdf$Additive + femdf$Age), type = "II", white.adjust = TRUE)
femAdd

## Coefficient covariances computed by hccm()
## Analysis of Deviance Table (Type II tests)
##
## Response: femdf$BMI
## Df F Pr(>F)
## femdf$Additive 2 2.7343 0.06517 .
## femdf$Age 1 132.3387 < 2e-16 ***
## Residuals 2134
## ---
## Signif. codes: 0 ‘***’ 0.001 ‘**’ 0.01 ‘*’ 0.05 ‘.’ 0.1 ‘ ’ 1

## BETA: female Additive
femAddmodel <- femAdd
femAddmodel <- sapply(femAddmodel, as.numeric)
lm(data.frame(scale(femAddmodel)))
## Call:
## lm(formula = data.frame(scale(femAddmodel)))
## Coefficients:
## (Intercept) F Pr..F.
## -0.5773502 -0.0005743 NA
confint(lm(data.frame(scale(femAddmodel))))
## 2.5 % 97.5 %
## (Intercept) NaN NaN
## F NaN NaN
## Pr..F. NA NA

## Female result: additive model not significant - and Age main effect very significant.


## BMI and Age DATA FOR TABLE 1: FEMALES

Additivedf <- data.frame(Additive, femdf[ , "BMI"], femdf[ , "Age"])
colnames(Additivedf) <- c("Additive", "BMI", "Age")
head(Additivedf)

Additivedf_0 <- Additivedf[Additivedf$Additive == 0, ]
head(Additivedf_0)
Additivedf_1 <- Additivedf[Additivedf$Additive == 1, ]
head(Additivedf_1)
Additivedf_2 <- Additivedf[Additivedf$Additive == 2, ]
head(Additivedf_2)

length(Additivedf_0$BMI)
length(Additivedf_1$BMI)
length(Additivedf_2$BMI)
length(Additivedf$BMI)

mean(Additivedf_0$BMI)
mean(Additivedf_1$BMI)
mean(Additivedf_2$BMI)
mean(Additivedf$BMI)

sd(Additivedf_0$BMI)
sd(Additivedf_1$BMI)
sd(Additivedf_2$BMI)
sd(Additivedf$BMI)

median(Additivedf_0$BMI)
median(Additivedf_1$BMI)
median(Additivedf_2$BMI)
median(Additivedf$BMI)

mad(Additivedf_0$BMI, constant = 1)
mad(Additivedf_1$BMI, constant = 1)
mad(Additivedf_2$BMI, constant = 1)
mad(Additivedf$BMI, constant = 1)

length(Additivedf_0$Age)
length(Additivedf_1$Age)
length(Additivedf_2$Age)
length(Additivedf$Age)

mean(Additivedf_0$Age)
mean(Additivedf_1$Age)
mean(Additivedf_2$Age)
mean(Additivedf$Age)

sd(Additivedf_0$Age)
sd(Additivedf_1$Age)
sd(Additivedf_2$Age)
sd(Additivedf$Age)

median(Additivedf_0$Age)
median(Additivedf_1$Age)
median(Additivedf_2$Age)
median(Additivedf$Age)

mad(Additivedf_0$Age, constant = 1)
mad(Additivedf_1$Age, constant = 1)
mad(Additivedf_2$Age, constant = 1)
mad(Additivedf$Age, constant = 1)


## Calculation of odds ratios:

## MALES AND FEMALES TOGETHER:
OverweightObesevec <- mydf1[(mydf1$BMI_GROUP == 3) | (mydf1$BMI_GROUP == 4), "rs67047829"]
OverweightObesevec
NormalUnderweightvec <- mydf1[(mydf1$BMI_GROUP == 1) | (mydf1$BMI_GROUP == 2), "rs67047829"]
NormalUnderweightvec
length(OverweightObesevec)
length(NormalUnderweightvec)
mydf1BMI_GROUP <- as.numeric(mydf1$BMI_GROUP)
mydf1BMI_GROUP
mydf1BMI_GROUPnoNA <- mydf1BMI_GROUP[!is.na(mydf1BMI_GROUP)]
length(mydf1BMI_GROUPnoNA)
length(OverweightObesevec) + length(NormalUnderweightvec)

OverweightObeseAAvec <- OverweightObesevec[OverweightObesevec == "AA"]
OverweightObeseAAvec
OverweightObeseGAGGvec <- OverweightObesevec[(OverweightObesevec == "GA") | (OverweightObesevec == "AG") | (OverweightObesevec == "GG")]
OverweightObeseGAGGvec

NormalUnderweightAAvec <- NormalUnderweightvec[NormalUnderweightvec == "AA"]
NormalUnderweightAAvec
NormalUnderweightGAGGvec <- NormalUnderweightvec[(NormalUnderweightvec == "GA") | (NormalUnderweightvec == "AG") | (NormalUnderweightvec == "GG")]
NormalUnderweightGAGGvec

length(OverweightObeseAAvec) + length(OverweightObeseGAGGvec) + length(NormalUnderweightAAvec) + length(NormalUnderweightGAGGvec)

OddsRatio <- (length(OverweightObeseAAvec) / length(OverweightObeseGAGGvec)) / (length(NormalUnderweightAAvec) / length(NormalUnderweightGAGGvec))
OddsRatio
## 0.4299128 - significant, see below:

TAB_MC <- matrix(c(length(OverweightObeseAAvec), length(OverweightObeseGAGGvec), length(NormalUnderweightAAvec), length(NormalUnderweightGAGGvec)),
 nrow = 2,
 dimnames = list(c("AA", "GA+GG"),
 c("OverweightObese", "NormalUnderweight")))
TAB_MC

or_fit <- oddsratio(TAB_MC)
or_fit
## $data
## OverweightObese NormalUnderweight Total
## AA 16 40 56
## GA+GG 2407 2587 4994
## Total 2423 2627 5050
##
## $measure
## NA
## odds ratio with 95% C.I. estimate lower upper
## AA 1.0000000 NA NA
## GA+GG 0.4326035 0.2341035 0.7611917
##
## $p.value
## NA
## two-sided midp.exact fisher.exact chi.square
## AA NA NA NA
## GA+GG 0.003242213 0.004324409 0.003461579
##
## $correction
## [1] FALSE
##
## attr(,"method")
## [1] "median-unbiased estimate & mid-p exact CI"

## MALES ONLY
Malesdf <- mydf1[mydf1$SEX == 1, ]
head(Malesdf)
OverweightMALEObesevec <- Malesdf[(Malesdf$BMI_GROUP == 3) | (Malesdf$BMI_GROUP == 4), "rs67047829"]
OverweightMALEObesevec
NormalUnderweightMALEvec <- Malesdf[(Malesdf$BMI_GROUP == 1) | (Malesdf$BMI_GROUP == 2), "rs67047829"]
NormalUnderweightMALEvec
length(OverweightMALEObesevec)
length(NormalUnderweightMALEvec)
MalesdfBMI_GROUP <- as.numeric(Malesdf$BMI_GROUP)
MalesdfBMI_GROUP
MalesdfBMI_GROUPnoNA <- MalesdfBMI_GROUP[!is.na(MalesdfBMI_GROUP)]
length(MalesdfBMI_GROUPnoNA)
length(OverweightMALEObesevec) + length(NormalUnderweightMALEvec)

OverweightMALEObeseAAvec <- OverweightMALEObesevec[OverweightMALEObesevec == "AA"]
OverweightMALEObeseAAvec
OverweightMALEObeseGAGGvec <- OverweightMALEObesevec[(OverweightMALEObesevec == "GA") | (OverweightMALEObesevec == "AG") | (OverweightMALEObesevec == "GG")]
OverweightMALEObeseGAGGvec

NormalUnderweightMALEAAvec <- NormalUnderweightMALEvec[NormalUnderweightMALEvec == "AA"]
NormalUnderweightMALEAAvec
NormalUnderweightMALEGAGGvec <- NormalUnderweightMALEvec[(NormalUnderweightMALEvec == "GA") | (NormalUnderweightMALEvec == "AG") | (NormalUnderweightMALEvec == "GG")]
NormalUnderweightMALEGAGGvec

length(OverweightMALEObeseAAvec) + length(OverweightMALEObeseGAGGvec) + length(NormalUnderweightMALEAAvec) + length(NormalUnderweightMALEGAGGvec)

OddsRatio <- (length(OverweightMALEObeseAAvec) / length(OverweightMALEObeseGAGGvec)) / (length(NormalUnderweightMALEAAvec) / length(NormalUnderweightMALEGAGGvec))
OddsRatio
## 0.4923726 - not significant - see below:
MALE_TAB_MC <- matrix(c(length(OverweightMALEObeseAAvec), length(OverweightMALEObeseGAGGvec), length(NormalUnderweightMALEAAvec), length(NormalUnderweightMALEGAGGvec)),
 nrow = 2,
 dimnames = list(c("AA", "GA+GG"),
 c("OverweightMALEObese", "NormalUnderweightMALE")))
MALE_TAB_MC

MALE_or_fit <- oddsratio(MALE_TAB_MC)
MALE_or_fit

## $data
## OverweightMALEObese NormalUnderweightMALE Total
## AA 8 24 32
## GA+GG 1027 1517 2544
## Total 1035 1541 2576
##
## $measure
## NA
## odds ratio with 95% C.I. estimate lower upper
## AA 1.000000 NA NA
## GA+GG 0.499083 0.2072639 1.07489
##
## $p.value
## NA
## two-sided midp.exact fisher.exact chi.square
## AA NA NA NA
## GA+GG 0.07711167 0.101396 0.07800849
##
## $correction
## [1] FALSE
##
## attr(,"method")
## [1] "median-unbiased estimate & mid-p exact CI"
