# Supplementary material for: rs67047829 genotypes of ERV3-1/ZNF117 are associated with lower body mass index in the Polish population
Source: Sci Rep. 2023 Oct 10;13:17118. doi: 10.1038/s41598-023-43323-3 (PMC10564729; doi:10.1038/s41598-023-43323-3)
Supplement: Supplementary file 5 — Supplementary Information 5. [file 41598_2023_43323_MOESM5_ESM.docx]

Supplementary_File_S5. Coding and results for Monte-Carlo Kruskal-Wallis power study.

## Article: "rs67047829 genotypes of ERV3-1/ZNF117 are associated with lower body mass index in the Polish population."

## Authors: Jeremy Clark*, Konrad Podsiadło*, Marta Sobalska-Kwapis, Błażej Marciniak, Kamila Rydzewska, Andrzej Ciechanowicz, Thierry van de Wetering, Dominik Strapagiel

## This file is modified from Supplementary File S8 in :

Clark, J.S.C., Kulig, P., Podsiadło, K. et al. Empirical investigations into Kruskal-Wallis power studies utilizing Bernstein fits, simulations and medical study datasets. Sci Rep 13, 2352 (2023). https://doi.org/10.1038/s41598-023-29308-2

## Monte Carlo KRUSKAL-WALLIS POWER STUDY TOOL - group-controlled ie. ratios of group sizes taken from "population" ratios.

## Assumes that entirely new data is to be used ie. that the data from the first study is NOT incorporated into the further study - OR for used for power estimation.

## Put the numeric data in place of the vector "Num".

## Put the factor data in place of the vector "Fac".

## In the example the 3 groups are: G1, G2, G3, and the numeric data for the groups are: Num1b, Num2b, Num3b.

## The coding is primarily designed to (1) give an estimate of sample size needed at a particular power. Alternatively, it can be used to (2) give the estimated power for a particular dataset.

## Please choose ONLY ONE of the following to be TRUE:

SAMPLE_SIZE_ESTIMATION <- FALSE ## TRUE or FALSE

POWER_ESTIMATION <- TRUE ## TRUE or FALSE

## 1. SAMPLE SIZE ESTIMATION. Set the following parameters as desired. Choose to set power or effect size or both (as minimums).

if (SAMPLE_SIZE_ESTIMATION == TRUE) {

power <- 0.8 ## usually 0.8 or 0.9. If not relevant, set to zero.

effectsize_limit <- 0 ## If effect size not relevant, set to zero.

alpha <- 0.01 ## usually 0.05 or 0.01. Two-tailed tests

nran <- 100 ## simulations at each step: large values e.g.>1000 are more accurate !

stepstart <- 1 ## usually starts at total sample size plus 1.

stepjump <- 1 ## usually jumps total sample size by 1 each time.

Nsteps <- 500 ## prevents unlimited execution.

## The following must be changed if the proportions to be selected in the future study are to be different from that in the sample. Example: POPpropA <- c(0.3, 0.2, 0.5)

POPpropA <- "not set" ## must be either "not set" or as example above

SlowTextplot <- FALSE ## small datasets TRUE = slow but elegant !

TurnOffMessages <- FALSE ## keep FALSE: messages or Textplot are recommended.

notice <- "Sample Size Estimation"

}

## 2. POWER ESTIMATION of a dataset. Set as indicated.

if (POWER_ESTIMATION == TRUE) {

power <- 0 ## set to zero

effectsize_limit <- 0 ## set to zero

alpha <- 0.05 ## choose alpha

nran <- 1000 ## large values e.g. >1000 are more accurate !

stepstart <- 1 ## small datasets leave at 1.

stepjump <- 1 ## small datasets leave at 1.

Nsteps <- 500 ## small datasets leave at 500.

POPpropA <- "not set" ## small datasets leave as "not set"

SlowTextplot <- FALSE ## small datasets TRUE = slow but elegant !

TurnOffMessages <- FALSE ## keep FALSE: messages or Textplot are recommended.

notice <- "Power Estimation"

}

## Libraries and miscellaneous functions:

## Note font is Arial (+ symbol) - which allows the number one, 1, to be distinguished from the letter l.

## install a library if necessary, then load:

options(timeout=1000)

ipak <- function(pkg){

new.pkg <- pkg[!(pkg %in% installed.packages()[, "Package"])]

if (length(new.pkg))

install.packages(new.pkg, dependencies = TRUE, repos="http://cran.r-project.org")

sapply(pkg, require, character.only = TRUE)

}

packages <- c("mlt", "outliers", "fitdistrplus", "logspline", "Matching", "actuar", "Rmisc", "ggplot2", "car", "plyr", "reshape2", "coin", "gplots", "LambertW", "nortest", "pwr", "gdata", "timeDate", "compiler", "data.table") ## References at end of file.

ipak(packages)

enableJIT(3) ## compiles for speed

na.pad <- function(x,len){ ## makes dataframes padded with NAs

x[1:len]

}

makePaddedDataFrame <- function(l,...){ ## needs list of vectors

maxlen <- max(sapply(l,length))

data.frame(lapply(l,na.pad,len=maxlen), drop = FALSE, ...)

}

## EXAMPLE DATA. Change so that Fac and Num refer to your data.

mypath <- "/Users/jeremyclark/Documents/aaJ Clark MACBOOK 2022 PUM/3 AA SUBMITTED PTCobesity Scientific Reports 140 pt/FIGS AND SUPP/"

dfSUP2 <- fread(paste0(mypath, "Supplementary_Table_S2_D.csv"))

dfSUP2 <- as.data.frame(dfSUP2)

colnames(dfSUP2) <- make.names(colnames(dfSUP2), unique = TRUE)

cols <- c("rs1421085", "rs1421085.1", "rs1558902", "rs1558902.1", "rs9939609", "rs9939609.1")

dfSUP <- dfSUP2[ , c("BMI", cols)]

dfSUP[ , cols] <- lapply(dfSUP[ , cols], as.factor)

dfSUP$BMI <- as.numeric(dfSUP$BMI)

dfSUP <- dfSUP[complete.cases(dfSUP[ , ]), ]

nrow(dfSUP)

rs1421085.gen <- paste0(dfSUP[ , "rs1421085"], dfSUP[ , "rs1421085.1"])

rs1558902.gen <- paste0(dfSUP[ , "rs1558902"], dfSUP[ , "rs1558902.1"])

rs9939609.gen <- paste0(dfSUP[ , "rs9939609"], dfSUP[ , "rs9939609.1"])

dfSUP$rs1421085.gen <- rs1421085.gen

dfSUP$rs1558902.gen <- rs1558902.gen

dfSUP$rs9939609.gen <- rs9939609.gen

rs1421085.CC <- dfSUP[dfSUP$rs1421085.gen %in% c("CC"), "rs1421085.gen"]

rs1421085.CT <- dfSUP[dfSUP$rs1421085.gen %in% c("CT", "TC"), "rs1421085.gen"]

rs1421085.TT <- dfSUP[dfSUP$rs1421085.gen %in% c("TT"), "rs1421085.gen"]

rs1421085.CC.BMI <- dfSUP[dfSUP$rs1421085.gen %in% c("CC"), "BMI"]

rs1421085.CT.BMI <- dfSUP[dfSUP$rs1421085.gen %in% c("CT", "TC"), "BMI"]

rs1421085.TT.BMI <- dfSUP[dfSUP$rs1421085.gen %in% c("TT"), "BMI"]

rs1558902.AA <- dfSUP[dfSUP$rs1558902.gen %in% c("AA"), "rs1558902.gen"]

rs1558902.AT <- dfSUP[dfSUP$rs1558902.gen %in% c("AT", "TA"), "rs1558902.gen"]

rs1558902.TT <- dfSUP[dfSUP$rs1558902.gen %in% c("TT"), "rs1558902.gen"]

rs1558902.AA.BMI <- dfSUP[dfSUP$rs1558902.gen %in% c("AA"), "BMI"]

rs1558902.AT.BMI <- dfSUP[dfSUP$rs1558902.gen %in% c("AT", "TA"), "BMI"]

rs1558902.TT.BMI <- dfSUP[dfSUP$rs1558902.gen %in% c("TT"), "BMI"]

rs9939609.AA <- dfSUP[dfSUP$rs9939609.gen %in% c("AA"), "rs9939609.gen"]

rs9939609.AT <- dfSUP[dfSUP$rs9939609.gen %in% c("AT", "TA"), "rs9939609.gen"]

rs9939609.TT <- dfSUP[dfSUP$rs9939609.gen %in% c("TT"), "rs9939609.gen"]

rs9939609.AA.BMI <- dfSUP[dfSUP$rs9939609.gen %in% c("AA"), "BMI"]

rs9939609.AT.BMI <- dfSUP[dfSUP$rs9939609.gen %in% c("AT", "TA"), "BMI"]

rs9939609.TT.BMI <- dfSUP[dfSUP$rs9939609.gen %in% c("TT"), "BMI"]

## Numeric data ie. measured values:

## Num1 <- rs1421085.CC.BMI

## Num1 <- rs1558902.AA.BMI

Num1 <- rs9939609.AA.BMI

median(Num1)

## Num2 <- rs1421085.CT.BMI

## Num2 <- rs1558902.AT.BMI

Num2 <- rs9939609.AT.BMI

median(Num2)

## Num3 <- rs1421085.TT.BMI

## Num3 <- rs1558902.TT.BMI

Num3 <- rs9939609.TT.BMI

median(Num3)

Num <- c(Num1, Num2, Num3)

## The factor vector indicates to which group the data belongs:

Fac1 <- rep("G1", length(Num1))

Fac2 <- rep("G2", length(Num2))

Fac3 <- rep("G3", length(Num3))

Fac <- c(Fac1, Fac2, Fac3)

## Numvar <- numeric_var("Num", support = c(min(Num), max(Num)), bounds = c(0, Inf))

mydf <- data.frame(Fac, Num)

colnames(mydf) <- c("Fac", "Num")

mydf$Fac <- factor(mydf$Fac, ordered = FALSE, levels = unique(mydf$Fac))

mydf <- mydf[order(mydf$Num), ]

head(mydf)

lapply(mydf, class)

length(mydf$Num)

## Split into Fac:

mydfG1 <- mydf[mydf$Fac %in% "G1", ]

head(mydfG1)

FacG1 <- mydfG1$Fac

NumG1 <- mydfG1$Num

length(NumG1)

mydfG2 <- mydf[mydf$Fac %in% "G2", ]

head(mydfG2)

FacG2 <- mydfG2$Fac

NumG2 <- mydfG2$Num

length(NumG2)

mydfG3 <- mydf[mydf$Fac %in% "G3", ]

head(mydfG3)

FacG3 <- mydfG3$Fac

NumG3 <- mydfG3$Num

length(NumG3)

length(NumG1) + length(NumG2) + length(NumG3)

length(mydf$Num)

POPpropB <- c(length(NumG1), length(NumG2), length(NumG3)) / ((length(NumG1) + length(NumG2) + length(NumG3)))

if (POPpropA == "not set") {

POPprop <- POPpropB

} else {

POPprop <- POPpropA

}

##__________________________________________________________

## KRUSKAL-WALLIS POWER STUDY - this assumes that a NEW STUDY is being done (ie. with no extension from the first subset).

#### ## KRUSKAL-WALLIS power study using assumed Bernstein distributions of samples using ratios of group sizes from the "population".

## A FUNCTION is created here which produces simulated data from the data above, and then performs a power study using the KRUSKAL-WALLIS test (rather than ANOVA) with group size proportions from the data (unless set otherwise).

## Use R {mlt} to produce simulated data:

if (SlowTextplot == TRUE) {

dev.new(width = 4, height = 4)

textplot(c("step = ", 0, ""), cex = 1)

}

## Note that group size proportions are taken from the data unless changed above:

myd3G1 <- mydf[mydf$Fac %in% "G1", ]

myd3G2 <- mydf[mydf$Fac %in% "G2", ]

myd3G3 <- mydf[mydf$Fac %in% "G3", ]

FacSAMG1 <- myd3G1$Fac

FacSAMG2 <- myd3G2$Fac

FacSAMG3 <- myd3G3$Fac

## Dataframes with duplicates of data:

mydfPP <- myd3G1

levels(mydfPP$Fac)[c(4, 5, 6)] <- c("PP", "PQ", "QQ")

mydfPP$Fac[mydfPP$Fac == "G1"] <- "PP"

mydfG1dup <- rbind(myd3G1, mydfPP)

mydfG1dup$Fac <- droplevels(mydfG1dup$Fac)

colnames(mydfG1dup)[colnames(mydfG1dup) == "Num"] <- "NumG1"

NumG1var <- numeric_var("NumG1", support =

c(min(mydfG1dup$NumG1), max(mydfG1dup$NumG1)))

mydfPQ <- myd3G2

levels(mydfPQ$Fac)[c(4, 5, 6)] <- c("PQ", "PQ", "QQ")

mydfPQ$Fac[mydfPQ$Fac == "G2"] <- "PQ"

mydfG2dup <- rbind( myd3G2, mydfPQ)

mydfG2dup$Fac <- droplevels(mydfG2dup$Fac)

colnames(mydfG2dup)[colnames(mydfG2dup) == "Num"] <- "NumG2"

NumG2var <- numeric_var("NumG2", support =

c(min(mydfG2dup$NumG2), max(mydfG2dup$NumG2)))

mydfQQ <- myd3G3

levels(mydfQQ$Fac)[c(4, 5, 6)] <- c("QQ", "PQ", "QQ")

mydfQQ$Fac[mydfQQ$Fac == "G3"] <- "QQ"

mydfG3dup <- rbind( myd3G3, mydfQQ)

mydfG3dup$Fac <- droplevels(mydfG3dup$Fac)

colnames(mydfG3dup)[colnames(mydfG3dup) == "Num"] <- "NumG3"

NumG3var <- numeric_var("NumG3", support =

c(min(mydfG3dup$NumG3), max(mydfG3dup$NumG3)))

bG1_Fac <- as.basis(~ Fac - 1, data = mydfG1dup)

bG2_Fac <- as.basis(~ Fac - 1, data = mydfG2dup)

bG3_Fac <- as.basis(~ Fac - 1, data = mydfG3dup)

levsG1dup <- as.character(unique(unlist(lapply(mydfG1dup$Fac, levels))))

levsG2dup <- as.character(unique(unlist(lapply(mydfG2dup$Fac, levels))))

levsG3dup <- as.character(unique(unlist(lapply(mydfG3dup$Fac, levels))))

ctmG1 <- ctm(response = Bernstein_basis(NumG1var, order = 4, ui = "increasing"), interacting = bG1_Fac, data = mydfG1dup)

ctmG2 <- ctm(response = Bernstein_basis(NumG2var, order = 4, ui = "increasing"), interacting = bG2_Fac, data = mydfG2dup)

ctmG3 <- ctm(response = Bernstein_basis(NumG3var, order = 4, ui = "increasing"), interacting = bG3_Fac, data = mydfG3dup)

### fit models

mltG1 <- mlt(ctmG1, data = mydfG1dup, optim = mltoptim(trace = TRUE, spg = list(maxit = 10000)))

mltG2 <- mlt(ctmG2, data = mydfG2dup, optim = mltoptim(trace = TRUE, spg = list(maxit = 10000)))

mltG3 <- mlt(ctmG3, data = mydfG3dup, optim = mltoptim(trace = TRUE, spg = list(maxit = 10000)))

#### STEP FUNCTION:

starttime = Sys.timeDate()

starttime

startsystime <- as.numeric(Sys.time(), digits=13)

ppvalueprop001 <- list(); nnsam <- list(); pvalue <- list(); pvalue001 <- list(); ppvalue <- list(); sG1 <- list(); sG2 <- list(); sG3 <- list(); ssG1 <- list(); ssG2 <- list(); ssG3 <- list(); ssNum <- list(); ssFac <- list(); sscombdf <- list(); kruskalss <- list(); ssFacG1 <- list(); ssFacG2 <- list(); ssFacG3 <- list(); ZscoreA <- list(); myeffectsizeA <- list(); mymineffectsizeB <- list(); medianeffectsizeB <- list(); medianeffectsizeA <- list(); tmpsG1 <- list(); tmpsG2 <- list(); tmpsG3 <- list(); ssAG1 <- list(); ssAG2 <- list(); ssAG3 <- list(); ssG11 <- list(); ssG21 <- list(); ssG31 <- list(); ssG12 <- list(); ssG22 <- list(); ssG32 <- list(); nsimG1 <- list(); nsimG2 <- list(); nsimG3 <- list();

for (i in seq(from = stepstart, to = Nsteps, by = stepjump)) {

ii <- i

nnsam[[i]] <- length(mydf$Num) + ii

nnsam[[i]]

Facprop <- POPprop ## proportions of group sizes.

nsimG1[[i]] <- floor(0.5 + (nnsam[[i]]*Facprop[[1]])) ## prop[[ 1 ]]

nsimG2[[i]] <- floor(0.5 + (nnsam[[i]]*Facprop[[2]])) ## prop[[ 2 ]]

nsimG3[[i]] <- nnsam[[i]] - nsimG1[[i]] - nsimG2[[i]]

sG1[[i]] <- list(); sG2[[i]] <- list(); sG3[[i]] <- list(); ssG1[[i]] <- list(); ssG2[[i]] <- list(); ssG3[[i]] <- list(); ssNum[[i]] <- list(); ssFac[[i]] <- list(); ppvalue[[i]] <- list(); sscombdf[[i]] <- list(); kruskalss[[i]] <- list(); ssFacG1[[i]] <- list(); ssFacG2[[i]] <- list(); ssFacG3[[i]] <- list(); ZscoreA[[i]] <- list(); myeffectsizeA[[i]] <- list(); medianeffectsizeA[[i]] <- c(); tmpsG1[[i]] <- list(); tmpsG2[[i]] <- list(); tmpsG3[[i]] <- list(); ssAG1[[i]] <- list(); ssAG2[[i]] <- list(); ssAG3[[i]] <- list(); ssG11[[i]] <- list(); ssG21[[i]] <- list(); ssG31[[i]] <- list(); ssG12[[i]] <- list(); ssG22[[i]] <- list(); ssG32[[i]] <- list(); pvalue001[[i]] <- list(); finaleffectsizes <- list(); finalZscores <- list(); finalpvalues <- list(); finalSampleSize <- list(); finalmineffectsize <- list(); finalmedianeffectsize <- list(); finalppropbelowalpha <- list();

for (j in 1:nran) {

jj <- j

sG1[[i]][[j]] <- simulate(mltG1, newdata = data.frame(Fac = unique(mydfG1dup$Fac)), nsim = 2*nsimG1[[i]]) ## gives ~4x number of simulations, reduced later

tmpsG1[[i]][[j]] <- sG1[[i]][[j]]

if (all(is.na(unlist(lapply(tmpsG1[[i]][[j]], '[[', 1))) == TRUE)) {

ssAG1[[i]][[j]] <- unlist(lapply(tmpsG1[[i]][[j]], '[[', 3))

} else {

ssG11[[i]][[j]] <- unlist(lapply(tmpsG1[[i]][[j]], '[[', 1))

ssG12[[i]][[j]] <- unlist(lapply(tmpsG1[[i]][[j]], '[[', 2))

ssAG1[[i]][[j]] <- c(ssG11[[i]][[j]], ssG12[[i]][[j]])

}

ssG1[[i]][[j]] <- ssAG1[[i]][[j]][is.finite(ssAG1[[i]][[j]])]

ssG1[[i]][[j]] <- ssG1[[i]][[j]][1:nsimG1[[i]]] ## gives length needed

ssFacG1[[i]][[j]] <-rep("G1", length(ssG1[[i]][[j]]))

sG2[[i]][[j]] <- simulate(mltG2, newdata = data.frame(Fac = unique(mydfG2dup$Fac)), nsim = 2*nsimG2[[i]]) ## gives ~4x number of simulations, reduced later

tmpsG2[[i]][[j]] <- sG2[[i]][[j]]

if (all(is.na(unlist(lapply(tmpsG2[[i]][[j]], '[[', 1))) == TRUE)) {

ssAG2[[i]][[j]] <- unlist(lapply(tmpsG2[[i]][[j]], '[[', 3))

} else {

ssG21[[i]][[j]] <- unlist(lapply(tmpsG2[[i]][[j]], '[[', 1))

ssG22[[i]][[j]] <- unlist(lapply(tmpsG2[[i]][[j]], '[[', 2))

ssAG2[[i]][[j]] <- c(ssG21[[i]][[j]], ssG22[[i]][[j]])

}

ssG2[[i]][[j]] <- ssAG2[[i]][[j]][is.finite(ssAG2[[i]][[j]])]

ssG2[[i]][[j]] <- ssG2[[i]][[j]][1:nsimG2[[i]]] ## gives length needed

ssFacG2[[i]][[j]] <-rep("G2", length(ssG2[[i]][[j]]))

sG3[[i]][[j]] <- simulate(mltG3, newdata = data.frame(Fac = unique(mydfG3dup$Fac)), nsim = 2*nsimG3[[i]]) ## gives ~4x number of simulations, reduced later

tmpsG3[[i]][[j]] <- sG3[[i]][[j]]

if (all(is.na(unlist(lapply(tmpsG3[[i]][[j]], '[[', 1))) == TRUE)) {

ssAG3[[i]][[j]] <- unlist(lapply(tmpsG3[[i]][[j]], '[[', 3))

} else {

ssG31[[i]][[j]] <- unlist(lapply(tmpsG3[[i]][[j]], '[[', 1))

ssG32[[i]][[j]] <- unlist(lapply(tmpsG3[[i]][[j]], '[[', 2))

ssAG3[[i]][[j]] <- c(ssG31[[i]][[j]], ssG32[[i]][[j]])

}

ssG3[[i]][[j]] <- ssAG3[[i]][[j]][is.finite(ssAG3[[i]][[j]])]

ssG3[[i]][[j]] <- ssG3[[i]][[j]][1:nsimG3[[i]]] ## gives length needed

ssFacG3[[i]][[j]] <-rep("G3", length(ssG3[[i]][[j]]))

#### Combine simulated data:

ssNum[[i]][[j]] <- as.numeric(c(ssG2[[i]][[j]], ssG1[[i]][[j]], ssG3[[i]][[j]]))

ssFac[[i]][[j]] <- as.factor(c(ssFacG2[[i]][[j]], ssFacG1[[i]][[j]], ssFacG3[[i]][[j]]))

sscombdf[[i]][[j]] <- matrix(ssNum[[i]][[j]], ssFac[[i]][[j]], nrow = length(ssNum[[i]][[j]]), ncol = 2)

colnames(sscombdf[[i]][[j]]) <- c("numeric", "factor")

sscombdf[[i]][[j]] <- sscombdf[[i]][[j]][is.finite(sscombdf[[i]][[j]][ , "numeric"]), ]

length(sscombdf[[i]][[j]][ , "numeric"])

kruskalss[[i]][[j]] <- kruskal.test(ssNum[[i]][[j]] ~ ssFac[[i]][[j]], data= sscombdf[[i]][[j]])

ppvalue[[i]][[j]] <- kruskalss[[i]][[j]]$p.value

ZscoreA[[i]][[j]] <- abs(qnorm(ppvalue[[i]][[j]]))

myeffectsizeA[[i]][[j]] <- ZscoreA[[i]][[j]] / sqrt(length(mydf$Num) + ii)

if (SlowTextplot == FALSE) {

if (TurnOffMessages == FALSE) {

message(paste(notice, "step: N + ", ii, " nran_Kruskal_tool = ", jj, ""))

}

} else {

textplot(c(notice, "step: N + ", ii, "nran_Kruskal_tool = ", jj, ""), cex = 1)

}

if (ppvalue[[i]][[j]] <= alpha) {

pvalue001[[i]][[j]] <- 1

} else {

pvalue001[[i]][[j]] <- 0

}

## Remove intermediates to save memory:

sG1[[i]][[j]] <- list(); sG2[[i]][[j]] <- list(); sG3[[i]][[j]] <- list(); kruskalss[[i]][[j]] <- list(); ssG1[[i]][[j]] <- list(); ssG2[[i]][[j]] <- list(); ssG3[[i]][[j]] <- list(); ssFacG1[[i]][[j]] <- list(); ssFacG2[[i]][[j]] <- list(); ssFacG3[[i]][[j]] <- list(); ssFac[[i]][[j]] <- list(); tmpsG1[[i]][[j]] <- list(); tmpsG2[[i]][[j]] <- list(); tmpsG3[[i]][[j]] <- list(); ssAG1[[i]][[j]] <- list(); ssAG2[[i]][[j]] <- list(); ssAG3[[i]][[j]] <- list(); ssG11[[i]][[j]] <- list(); ssG21[[i]][[j]] <- list(); ssG31[[i]][[j]] <- list(); ssG12[[i]][[j]] <- list(); ssG22[[i]][[j]] <- list(); ssG32[[i]][[j]] <- list();

} ## from for (j in 1:nran) {

pvalue[[i]] <- as.vector(unlist(ppvalue[[i]]))

pvalue001[[i]] <- as.vector(unlist(pvalue001[[i]]))

ZscoreA[[i]] <- as.vector(unlist(ZscoreA[[i]]))

myeffectsizeA[[i]] <- as.vector(unlist(myeffectsizeA[[i]]))

ppvalueprop001[[i]] <- sum(pvalue001[[i]] == 1) / nran

medianeffectsizeA[[i]] <- median(unlist(myeffectsizeA[[i]]), na.rm = TRUE)

if(ppvalueprop001[[i]] >= power) {

if(medianeffectsizeA[[i]] >= effectsize_limit) {

finaleffectsizes[[i]] <- myeffectsizeA[[i]]

finalZscores[[i]] <- ZscoreA[[i]]

finalpvalues[[i]] <- pvalue[[i]]

finalSampleSize[[i]] <- nnsam[[i]]

finalmineffectsize[[i]] <- min(myeffectsizeA[[i]], na.rm = TRUE)

finalmedianeffectsize[[i]] <- medianeffectsizeA[[i]]

finalppropbelowalpha[[i]] <- ppvalueprop001[[i]]

break

} ## from if(ppvalueprop001

} ## from if(medianeffectsizeA

## Remove intermediates to save memory:

sG1[[i]] <- list(); sG2[[i]] <- list(); sG3[[i]] <- list(); kruskalss[[i]] <- list(); ssG1[[i]] <- list(); ssG2[[i]] <- list(); ssG3[[i]] <- list(); ssFacG1[[i]] <- list(); ssFacG2[[i]] <- list(); ssFacG3[[i]] <- list(); ssFac[[i]] <- list(); ssNum[[i]] <- list(); sscombdf[[i]] <- list(); sscombdf[[i]] <- list(); ppvalue[[i]] <- list(); tmpsG1[[i]] <- list(); tmpsG2[[i]] <- list(); tmpsG3[[i]] <- list(); ssAG1[[i]] <- list(); ssAG2[[i]] <- list(); ssAG3[[i]] <- list(); ssG11[[i]] <- list(); ssG21[[i]] <- list(); ssG31[[i]] <- list(); ssG12[[i]] <- list(); ssG22[[i]] <- list(); ssG32[[i]] <- list(); nsimG1[[i]] <- list(); nsimG2[[i]] <- list(); nsimG3[[i]] <- list();

} ## from for i

## End of function for Kruskal power study for data.

## The following gives the effect sizes for the step with power => power

finaleffectsizes <- unlist(finaleffectsizes)

finalZscores <- unlist(finalZscores)

finalpvalues <- unlist(finalpvalues)

finalSampleSize <- unlist(finalSampleSize)

finalmineffectsize <- unlist(finalmineffectsize)

finalmedianeffectsize <- unlist(finalmedianeffectsize)

finalppropbelowalpha <- unlist(finalppropbelowalpha)

notice

finaleffectsizes

finalZscores

finalpvalues

finalSampleSize ## estimated SAMPLE SIZE needed

finalmineffectsize ## estimated MINIMUM EFFECT SIZE

finalmedianeffectsize ## estimated MEDIAN EFFECT SIZE

finalppropbelowalpha ## MEASURED POWER

notice

finishtime = Sys.timeDate()

finishtime

timeelapsed = finishtime - starttime

timeelapsed

finishsystime <- as.numeric(Sys.time(), digits=13)

systimeelapsed <- finishsystime - startsystime

systimeelapsed

## REFERENCES.

## References in same order as in packages vector:

## 1. Hothorn, T. mlt: Most Likely Transformations. (2017).at <https://CRAN.R-project.org/package=mlt>

## 2. Komsta, L. outliers: Tests for outliers. (2011).at <https://CRAN.R-project.org/package=outliers>

## 3. Delignette-Muller, M. L. & Dutang, C. fitdistrplus: An R Package for Fitting Distributions. Journal of Statistical Software 64, 1–34 (2015).

## 4. Kooperberg, C. logspline: Logspline Density Estimation Routines. (2016).at <https://CRAN.R-project.org/package=logspline>

## 5. Sekhon, J. S. Multivariate and propensity score matching software with automated balance optimization: the matching package for R. (2011).

## 6. Fernandez, E. S. Johnson: Johnson Transformation. (2014).at <https://CRAN.R-project.org/package=Johnson>

## 7. Dutang, C., Goulet, V. & Pigeon, M. actuar: An R Package for Actuarial Science. Journal of Statistical Software 25, 38 (2008).

## 8. Hope, R. M. Rmisc: Rmisc: Ryan Miscellaneous. (2013).at <https://CRAN.R-project.org/package=Rmisc>

## 9. Wickham, H. ggplot2: Elegant Graphics for Data Analysis. (Springer-Verlag New York: 2009).at <http://ggplot2.org>

## 10. Fox, J. & Weisberg, S. An R Companion to Applied Regression. (Sage: Thousand Oaks CA, 2011).at <http://socserv.socsci.mcmaster.ca/jfox/Books/Companion>

## 11. Wickham, H. The Split-Apply-Combine Strategy for Data Analysis. Journal of Statistical Software 40, 1–29 (2011).

## 12. Wickham, H. Reshaping Data with the reshape Package. Journal of Statistical Software 21, 1–20 (2007).

## 13. Hothorn, T., Hornik, K., Van De Wiel, M. A., Zeileis, A. & others Implementing a class of permutation pests: the coin package. (2008).

## 14. Warnes, G. R. et al. gplots: Various R Programming Tools for Plotting Data. (2016).at <https://CRAN.R-project.org/package=gplots>

## 15. Goerg, G. LambertW: An R package for Lambert W$\times$ F Random Variables. R package version 0.6 4, (2016).

## 16. Gross, J. & Ligges, U. nortest: Tests for Normality. (2015).at <https://CRAN.R-project.org/package=nortest>

## 17. Champely, S. pwr: Basic Functions for Power Analysis. (2017).at <https://CRAN.R-project.org/package=pwr>

## 18. Warnes, G. R. et al. gdata: Various R Programming Tools for Data Manipulation. (2017).at <https://CRAN.R-project.org/package=gdata>

## 19. Team, R. C. et al. timeDate: Rmetrics - Chronological and Calendar Objects. (2015).at <https://CRAN.R-project.org/package=timeDate>

## 20. R Core Team (2020). R: A language and environment for statistical computing. R Foundation for Statistical Computing, Vienna, Austria. URL https://www.R-project.org/.

"rs1421085"

## > finalSampleSize ## estimated SAMPLE SIZE needed

## [1] 5095

## > finalmineffectsize ## estimated MINIMUM EFFECT SIZE

## [1] 0.001096383

## > finalmedianeffectsize ## estimated MEDIAN EFFECT SIZE

## [1] 0.03900325

## > finalppropbelowalpha ## MEASURED POWER

## [1] 0.855

## > notice

## [1] "Power Estimation"

"rs1558902"

## > finalSampleSize ## estimated SAMPLE SIZE needed

## [1] 5094

## > finalmineffectsize ## estimated MINIMUM EFFECT SIZE

## [1] 0.0004586488

## > finalmedianeffectsize ## estimated MEDIAN EFFECT SIZE

## [1] 0.04021509

## > finalppropbelowalpha ## MEASURED POWER

## [1] 0.866

## > notice

## [1] "Power Estimation"

"rs9939609"

## > finalSampleSize ## estimated SAMPLE SIZE needed

## [1] 5092

## > finalmineffectsize ## estimated MINIMUM EFFECT SIZE

## [1] 4.685681e-05

## > finalmedianeffectsize ## estimated MEDIAN EFFECT SIZE

## [1] 0.03698448

## > finalppropbelowalpha ## MEASURED POWER

## [1] 0.821

## > notice

## [1] "Power Estimation"
